# Generation of an integrated *Hieracium* genomic and transcriptomic resource enables exploration of small RNA pathways during apomixis initiation

**DOI:** 10.1186/s12915-016-0311-0

**Published:** 2016-10-06

**Authors:** David S. Rabiger, Jennifer M. Taylor, Andrew Spriggs, Melanie L. Hand, Steven T. Henderson, Susan D. Johnson, Karsten Oelkers, Maria Hrmova, Keisuke Saito, Go Suzuki, Yasuhiko Mukai, Bernard J. Carroll, Anna M. G. Koltunow

**Affiliations:** 1 Commonwealth Scientific and Industrial Research Organisation Agriculture and Food, Private Bag 2, Glen Osmond, South Australia 5064 Australia; 2 Commonwealth Scientific and Industrial Research Organisation Agriculture and Food, Bellenden Street, Crace, Australian Capital Territory 2911 Australia; 3 Australian Centre for Plant Functional Genomics, University of Adelaide PMB 1, Glen Osmond, South Australia 5064 Australia; 4 Division of Natural Science, Osaka Kyoiku University, Osaka, 582-8582 Japan; 5 School of Chemistry and Molecular Biosciences, University of Queensland, St. Lucia, Queensland 4072 Australia

## Abstract

**Background:**

Application of apomixis, or asexual seed formation, in crop breeding would allow rapid fixation of complex traits, economizing improved crop delivery. Identification of apomixis genes is confounded by the polyploid nature, high genome complexity and lack of genomic sequence integration with reproductive tissue transcriptomes in most apomicts.

**Results:**

A genomic and transcriptomic resource was developed for *Hieracium* subgenus *Pilosella* (Asteraceae) which incorporates characterized sexual, apomictic and mutant apomict plants exhibiting reversion to sexual reproduction. Apomicts develop additional female gametogenic cells that suppress the sexual pathway in ovules. Disrupting small RNA pathways in sexual *Arabidopsis* also induces extra female gametogenic cells; therefore, the resource was used to examine if changes in small RNA pathways correlate with apomixis initiation. An initial characterization of small RNA pathway genes within *Hieracium* was undertaken, and ovary-expressed *ARGONAUTE* genes were identified and cloned. Comparisons of whole ovary transcriptomes from mutant apomicts, relative to the parental apomict, revealed that differentially expressed genes were enriched for processes involved in small RNA biogenesis and chromatin silencing. Small RNA profiles within mutant ovaries did not reveal large-scale alterations in composition or length distributions; however, a small number of differentially expressed, putative small RNA targets were identified.

**Conclusions:**

The established *Hieracium* resource represents a substantial contribution towards the investigation of early sexual and apomictic female gamete development, and the generation of new candidate genes and markers. Observed changes in small RNA targets and biogenesis pathways within sexual and apomictic ovaries will underlie future functional research into apomixis initiation in *Hieracium*.

**Electronic supplementary material:**

The online version of this article (doi:10.1186/s12915-016-0311-0) contains supplementary material, which is available to authorized users.

## Background

Apomixis describes a suite of reproductive processes in flowering plants that result in asexual seed formation, and subsequently give rise to progeny which are clones of the maternal parent [1]. Contrary to the sexual pathway, wherein the diversity of progeny is derived from meiotic recombination during gamete formation followed by parental gamete fusion and embryogenesis at fertilization, apomixis avoids meiosis during female gamete development, and embryogenesis initiates without fertilization. Use of apomixis in plant breeding could economize and speed delivery of new plant varieties by preserving unlinked complex traits, such as heterosis in hybrid crops, within a single seed generation. As apomixis is not evident in major seed crops, isolation of genes from apomictic model species with the intention to transfer them to crops has been a major focus of research.

Most apomictic species are polyploids, often with large genomes, and evidence suggests apomixis is generally conferred by dominant loci in most examined species [2, 3]. Apomictic loci are often found to be associated with highly repetitive chromosomal regions where recombination is suppressed, and additional loci also appear to influence the penetrance of the apomixis phenotype [1, 3, 4]. To date, genes within apomictic loci which are capable of conferring apomixis phenotypes are largely unknown, with the exception of a recently identified gene from apomictic *Pennisetum* that stimulates fertilization-independent embryogenesis [5].

Identification of genes regulating apomixis has been hindered due to limited genomic and transcriptomic resources available in apomict model species. Several transcriptomic analyses in apomictic species have been published to date, including serial analysis of gene expression (SAGE), microarray approaches [6–8], and more recently de novo sequencing [9, 10]. However, the generation of genomic resources within these species has generally been neglected, and an integrated resource combining genomic and transcriptomic data in any one apomictic species has not yet been realized. Furthermore, genome assembly and annotation has been difficult due to the high complexity of polyploid genomes in apomicts, and de novo transcriptomic assemblies are likely to underrepresent the diversity of transcripts actually present due to the collapsing of homologous genes with high sequence similarity into chimeric contigs [11, 12].

*Hieracium* subgenus *Pilosella* species within the Asteraceae contain both obligate sexual and facultative apomictic species where apomixis is not fully penetrant [13, 14]. Within apomictic species the trait is dominant, and apomixis occurs via aposporous embryo sac formation (described below). Apomictic *Hieracium* species are also amongst the few known apomicts capable of both fertilization-independent embryo and endosperm development during seed formation [15–18]. Recombination at apomixis loci within the *Pilosella* subgenus of *Hieracium* does occur at low rates when crossed with sexual species, which in combination with a collection of apomixis deletion mutants, available bacterial artificial chromosome (BAC) libraries and expressed sequence markers, has aided in the generation of a genetic linkage map [16–20]. Coupled with a short lifecycle and an established transformation capability, these species have been developed into a valuable molecular and genetic model for the analysis of apomixis. Currently, the closest phylogenetic relative to *Hieracium* with a comprehensively sequenced and annotated genome is the tomato (*Solanum lycopersicum*), within the Solanaceae, which reproduces exclusively through typical sexual seed formation pathways [21]. A substantial expressed sequence tag (EST) database from a closer relative within the Asteraceae, lettuce (*Lactuca sativa*), also an obligate sexual, is publicly available together with a high-density genetic map [22]; however, functional annotation within the lettuce EST resource is limited. The availability of a *Hieracium* genomic resource coupled with temporally staged ovary transcriptomes, where the events of apomixis take place, could significantly accelerate the isolation of apomixis genes and loci.

**Fig. 1:**
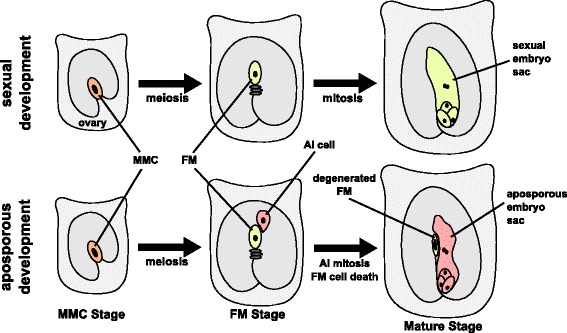
Sexual and aposporous gametophyte development in *Hieracium. Hieracium* ovaries contain a single ovule. Specification of the megaspore mother cell (*MMC*) occurs at the MMC stage in ovules of both sexual (*top*) and aposporous (*bottom*) species. At the functional megaspore (*FM*) stage, meiosis has occurred and aposporous ovules contain aposporous initial (*AI*) cells in addition to a meiotic tetrad where the FM is expanding. Within sexual ovules, the FM undergoes mitosis, generating a meiotically reduced gametophyte. Within aposporous ovules, the AI cell initiates a mitotic program to generate an unreduced aposporous gametophyte, and the adjacent sexual tetrad with enlarging FM degrades. Within aposporous ovules, the aposporous embryo sac will initiate fertilization-independent embryo and endosperm development to generate a clonal seed, whereas sexual ovules require fertilization (not shown). For additional details of sexual and apomictic development, refer to the text

Within aposporous species of *Hieracium*, specification of the megaspore mother cell (MMC), meiosis and selection of the functional megaspore (FM) occur as found during typical polygonum-type female gamete development in sexual plant species (Fig. 1) [23]. However, following the initiation of meiosis, one or more cells from sporophytic tissue, termed aposporous initial (AI) cells, enlarge near the cells undergoing the events of meiosis and megaspore selection in the majority of ovules (Fig. 1). One of the AI cells initiates a mitotic gametophyte program which results in the formation of an aposporous gametophyte and concurrently leads to degeneration of the developing sexual female gametophyte (Fig. 1). Inhibiting meiosis within the MMC prevents AI cell initiation, suggesting that ovule cues associated with activation and progression of meiosis may stimulate AI cell development [18]. Transcriptomic analysis of microdissected AI cells suggests that enlarging AI cells do not express key meiosis genes [9]. The egg and central cell in the aposporous embryo sac then undergo fertilization-independent embryo and endosperm development [24].

A collection of gamma irradiated deletion mutants within the aposporous *Hieracium* species *H. praealtum* (R35) identified two independent and dominant loci, *LOSS OF APOMEIOSIS* (*LOA*) and *LOSS OF PARTHENOGENESIS* (*LOP*), which are required for AI cell initiation and fertilization-independent seed development, respectively [16, 18]. Deletion mutants lacking *LOA* fail to initiate AI cell development, and therefore sexual gametophytic development progresses to completion. Sexual gametophytes that inherit a functional *LOP* locus, in the absence of *LOA*, can still initiate fertilization-independent seed formation resulting in plants with reduced ploidy relative to the parent. Deletion mutants lacking *LOP* generate unreduced gametophytes mitotically through AI cells. However, they are not capable of undergoing fertilization-independent embryo or endosperm development and therefore require fertilization by a male gamete to initiate seed development, resulting in plants with increased ploidy. Deletion of both loci results in a complete reversion to sexual reproduction, indicating that sexual reproduction is the default state in the apomict, and the apomixis phenotype in R35 is superimposed upon and suppresses normal sexual development [18]. The model for apomixis regulation in subgenus *Pilosella* currently posits that the initiation of meiosis during sexual megaspore development activates *LOA,* enabling specification of an apomictic lineage with AI cell formation. The AI cell recruits genes normally involved in the mitotic events of sexual female gametogenesis, and as it undergoes aposporous embryo sac formation the adjacent sexual gametophyte is actively suppressed. *LOP* later functions together with other identified seed initiation loci to either de-repress or activate genes involved in embryo and endosperm formation within the aposporous embryo sac, overcoming the requirement for fertilization [25].

A cytological analysis with *LOA*-specific probes indicates *LOA* is localized within a hemizygous, repeat-rich chromosomal region, and molecular markers linked to *LOA* and *LOP* have been shown to be conserved in additional *Hieracium* species, including *H. piloselloides* (D36) and *H. caespitosum* [17, 19]. While much has been learned regarding the genomic region surrounding both *LOA* and *LOP*, very little is known about the specific loci themselves, or any putative genes contained within them.

Interestingly, recent studies in sexual plants indicate that species-specific disruptions in small RNA biogenesis and function can cause apomixis-like initiation of female gametogenic development. In *Arabidopsis*, mutations affecting *ARGONAUTE9* (*AGO9*) cause formation of additional gametic precursor cells in the ovule, which is reminiscent of the apospory phenotype [26]. In *Zea mays* mutations that affect *ZmAGO4d*/*AGO104*, the sexual MMC avoids meiosis and forms unreduced embryo sacs reminiscent of apomictic development in diplosporous species [27]. Both *AGO* genes are involved in the maintenance of non-CG DNA methylation through the RNA-dependent DNA methylation (RdDM) pathway [28]. Additional genes involved in the biogenesis of small RNAs have also been shown to induce the formation of extra cells with gametogenic potential in *Arabidopsis* mutants. Mutations in *RNA-DEPENDENT RNA POLYMERASE2* (*RDR2*) and *DICER-LIKE3* (*DCL3*), genes required for the production of 24-nt small interfering RNAs (siRNAs) as part of the RdDM pathway, as well as *RDR6* and *SUPPRESSOR OF GENE SILENCING3* (*SGS3*), which are required for the production of 21-nt siRNAs and trans-acting siRNAs (tasiRNAs), all induce formation of extra gametic precursor cells [26]. Small RNAs may also be involved in sexual gametophyte development, as evidenced by a semi-dominant mutant affecting *AGO5* in *Arabidopsis*, which fails to initiate megagametogenesis following selection of the post-meiotic FM [29]. While these data indicate that a number of genes involved in small RNA biogenesis or function are required for specification and development of female gametogenesis within these normally sexual species, whether small RNAs might also be involved in the regulation of apomictic pathways within naturally apomictic species is currently unknown.

In this paper, we introduce a genomic survey sequence of D18, a dihaploid apomictic plant derived from a rare meiotically reduced egg that underwent parthenogenesis within the tetraploid apomict *H. piloselloides* (D36). D18 has been demonstrated to contain the *LOA*-associated chromosome and markers [17, 30]. We used the D18 genomic resource to investigate apomixis initiation through the analysis of transcriptomes and small RNA profiles in ovaries isolated from sexual and apomictic plants, and provide an initial characterization of small RNA pathway genes within *Hieracium*. Comparisons of ovary transcriptomes from apomictic R35 and two R35 deletion mutants unable to initiate apomixis (*m115* and *m134*) identified differentially expressed genes in common between the two mutants to be enriched for gene ontology (GO) terms associated with small RNA biogenesis and chromatin silencing via the RdDM pathway. However, for genes associated with these GO terms, the direction of their fold change was not conserved between the two mutants. Comparisons of small RNA length distributions between R35 and the *m115* or *m134* deletion mutant did not reveal any large-scale alterations, although an alignment of small RNAs to D18 genomic contigs identified a small number of putative differentially targeted genes within the two mutants. These data provide an extensive integrated platform to aid in the identification of apomixis-related genes and the investigation of the role of small RNAs during asexual gametophyte development.

## Results

### Generation of an apomictic *Hieracium* genomic and transcriptomic resource

In order to maximize genome sequence coverage in apomictic *Hieracium*, we generated a de novo assembly from genomic DNA isolated from the dihaploid apomict D18, which consists of two reduced subgenomes as compared to the predominantly tetraploid *Hieracium* plants we have previously characterized (Table 1). The predicted genome size of D18 is 3.6 Gb (1C value) [31], and the resulting de novo D18 genomic assembly consisted of 2.97 Gb of unordered contigs (Additional file 1: Table S1). A filtered set of 0.5 million contigs ≥2 Kb was used in subsequent analyses, totalling 2.92 Gb in length, with an N50 of 7.3 Kb. Markers that have been previously shown to be linked to *LOA* were identified within the resulting contigs; however, a single contig spanning all linked markers was not assembled, likely due to the repeat-rich context typically found at the *LOA* locus [17, 19].

**Table 1:** Summary of genotypes and phenotypes of *Hieracium* plants used in the analysis and nucleic acids examined

Species (genotype)	Genetic constitution^a^	Phenotype^b^	MMC stage ovaries	FM stage ovaries	Leaves
*H. praealtum* (R35)	2*n* = 4x-1 = 35	Apomict *LOA/LOP*	mRNA sRNA	mRNA sRNA	sRNA
*H. piloselloides* (D36)	2*n* = 4x = 36	Apomict *LOA/LOP*	mRNA sRNA	mRNA sRNA	nd
*H. piloselloides* (D18)	2n = 2x = 18^c^	Apomict *LOA/LOP*	nd	nd	DNA
*H. praealtum* (*m115*)	2*n* = 4x-1 = 35^d,e^	Sexual *loa/lop*	mRNA sRNA	mRNA sRNA	sRNA
*H. praealtum* (*m134*)	2*n* = 4x = 36^e^	Sexual^f^ *loa/LOP*	mRNA sRNA	mRNA sRNA	sRNA
*H. pilosella* (P36)	2*n* = 4x = 36	Sexual	mRNA sRNA	mRNA sRNA	nd

*nd* not done

^a^Indicates ploidy of the sporophyte, and the chromosome number

^b^ *LOA* and *LOP* indicate apospory and autonomous seed formation are evident, respectively; *loa* and *lop* indicate their phenotypic absence

^c^D18, a dihaploid derived from D36 (see introduction)

^d^The chromosome number of *m115* is presumed to be the same as R35

^e^ *m115* and *m134* are derived from gamma-ray mutagenesis of R35

^f^Gametophytes in *m134* ovules are sexually derived, but are still capable of autonomous seed development

To complement the genomic assembly, whole ovary transcriptomes were generated from the apomicts D36 and *H. praealtum* (R35), a sexual species *H. pilosella* (P36) and two *LOA* deletion mutants derived from gamma-ray mutagenesis of R35 (*m115* and *m134;*see Table 1). While both *m115* and *m134* are aposporous mutants that have lost the ability to form AI cells and only form sexual female gametophytes, *m115* is a complete sexual revertant because it has also lost *LOP*, whereas *m134* retains *LOP* function [18]. Although *m134* has lost the apospory phenotype, it retains the repeat-rich region surrounding *LOA*, and metaphase chromosome spreads indicate that *m134* has 36 chromosomes in contrast to the 35 chromosomes evident in the progenitor R35 (Additional file 2: Figure S1). Chromosome numbers in the set of *Hieracium* deletion mutants range from 34–36, as in addition to inducing deletions and chromosomal rearrangements, ionizing radiation is known to result in gain or loss of chromosomes [19, 32, 33].

Given our focus on gene expression during apomixis initiation, we analysed transcriptomes and small RNAs isolated from whole ovaries of sexual and apomictic plants at two developmental stages characterized by distinct sexual events. The earlier megaspore mother cell (MMC) stage consisted of ovaries wherein the ovules contained MMCs undergoing meiosis (Fig. 1). The later stage, designated the functional megaspore (FM) stage, consisted of ovaries wherein the ovules contained a specified functional megaspore (Fig. 1). Within MMC stage ovules of apomicts, AI cell initiation is not yet visible, whereas within the FM stage, enlarged AI cells are evident in the majority of ovules (see ‘Methods’). The independent de novo transcriptomic assemblies for each stage and genotype generated ranged in size from 46 to 110 thousand contigs, and the N50 of contig lengths varied between 1.4 and 1.7 Kb (Additional file 1: Table S1).

An *abinitio* gene model prediction analysis of the assembled genomic D18 contigs yielded 619,677 predicted gene models that were further annotated for both nucleotide and predicted coding sequence (CDS) correspondence to known gene sequence sets from *Arabidopsis* (TAIR10), tomato (ITAG2.4), lettuce ESTs (Compositae Genome Project: http://compgenomics.ucdavis.edu/) and several other plant species (Additional file 1: Table S1). The assembled genomic D18 contigs and predicted gene models were assessed for sequence diversity to provide an estimate of gene copy number and heterozygosity. Based on blast alignments, 32.8 % of predicted D18 gene models have a copy number of 2 or less, and 53 % of D18 genomic sequences were within 2 single-nucleotide polymorphisms (SNPs) per 100 bases of each other. We also assessed the genomic and transcriptomic assemblies for coverage of known tomato cDNAs and lettuce ESTs to provide an estimate of the gene coverage present in the resource. The average coverage of tomato cDNAs from all assemblies was observed to be 38.2 %, and the average coverage of lettuce ESTs and lettuce unigenes was 67.0 % and 45.3 %, respectively, reflecting the closer phylogenetic history of *Hieracium* and lettuce (Additional file 1: Table S1). The average number of D18 CDSs that could be mapped to each lettuce EST was 1.8, and to each unigene was 2.8. By comparison, on average 1.8 lettuce ESTs could be mapped to each lettuce unigene. The proportion of assembled *Hieracium* transcript contigs aligning to the D18 assembly averaged 74 % for the transcriptomes derived from the R35 backgrounds, and 83 % for the D36 transcriptomic assemblies (Additional file 1: Table S1). The genomic and transcriptomic data have been integrated into a publically available genome browser, along with a sequence blast server, accessible as described in the ‘Availability of data and materials’ section.

### Expression of sexual pathways in sexual and apomictic *Hieracium* ovaries during early developmental stages

Sexual gametogenesis initiates and proceeds to the functional megaspore stage within all ovules of the sexual and apomictic plants examined in our transcriptomes. We first attempted to gain an indication of the variation in sexual pathway gene expression between genotypes and across the two developmental stages, in addition to exploring the sensitivity of sexual transcript detection in whole ovaries (Fig. 1; Table 1). To do this we accessed reproductive functional gene categories in *Arabidopsis*, an obligate sexual species, using VirtualPlant [34]. These included genes involved in male and female gametophyte development, meiosis, cell fate specification and multiple associated reproductive processes (Additional file 1: Table S2). This subset of 1347 *Arabidopsis* cDNAs was associated with D18 predicted gene models using blastp, and transcriptome reads from R35, D36, P36, *m115* and *m134* ovary samples at MMC and FM stages were aligned to the D18 gene models. Of the initial 1347 gene models, 904 could be associated with transcriptome reads. In general, most genes displayed stable expression across the samples studied (Additional file 3).

These analyses detected the expression of genes typically found in low levels in specific *Arabidopsis* ovule cell types. One example is an ortholog of *WUSCHEL* (At2g17950), a homeobox gene shown to have highly cell-specific expression during specification of stem cell niches, as well as the initiation of integument development in ovules and specification of the MMC [35–38]. This supports the sensitivity of reproductive transcript detection represented by relatively few cell types in the whole ovary samples.

**Fig. 2:**
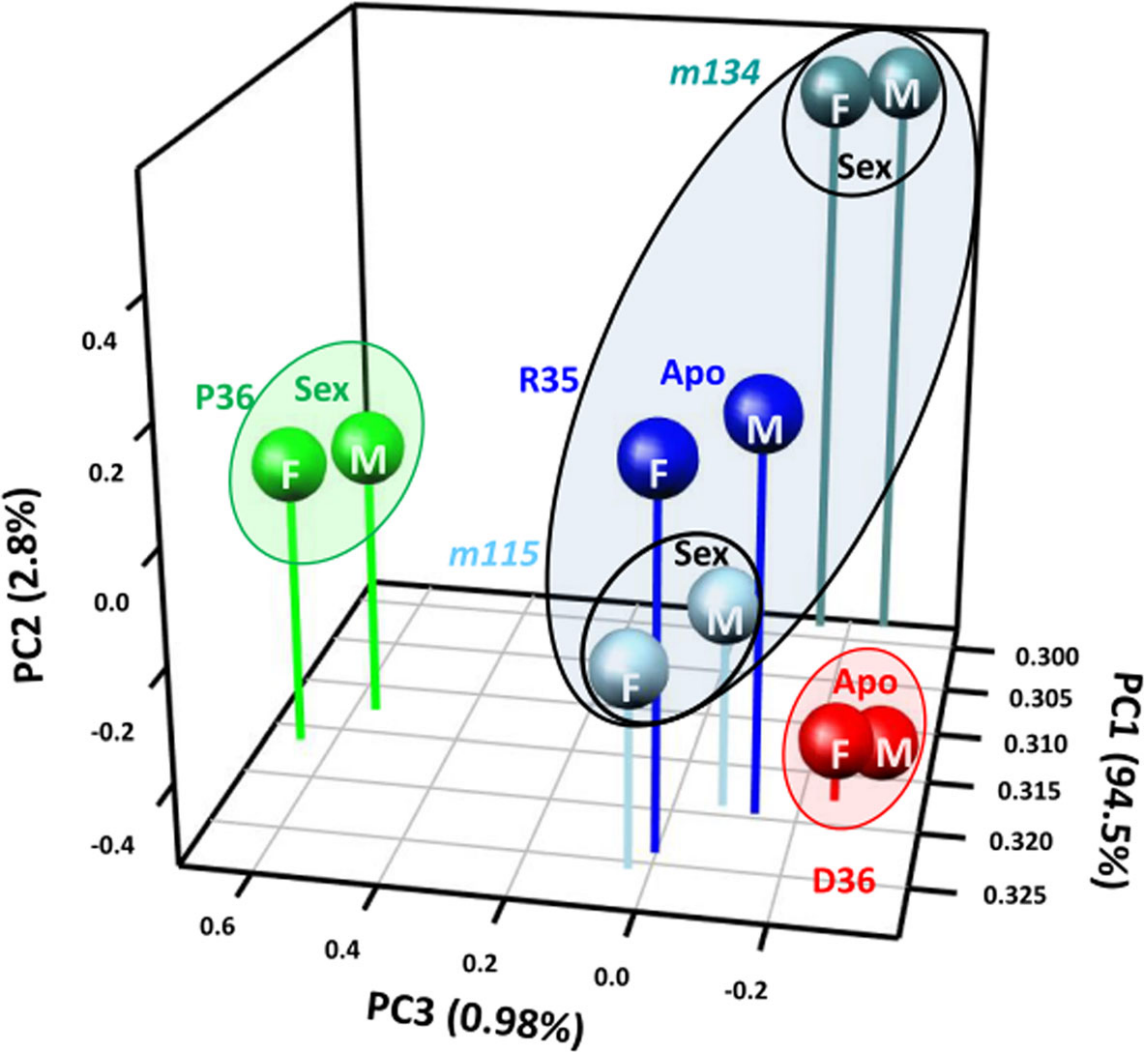
Principal component analysis of sexual pathway genes in *Hieracium* ovaries. Principal component analysis of 904 sexual pathway genes in apomictic R35 and D36, sexual P36, *m115* and *m134,* apomict mutants derived from R35, at both the MMC (marked as *M*) and FM (marked as *F*) stages. Samples are largely separated by genotype, with developmental stage contributing less to variation. *Sex* sexual phenotype, *Apo* apomictic phenotype. Percentage variation for each principal component is shown at each axis

A principal component analysis (PCA) of the 904 *Hieracium* gene models and their expression across all samples highlighted greater separation between the three species represented by P36, D36 and R35, and less distinction between apomicts R35 and D36. Within each genotype, little separation was observed between the two developmental stages. The apomict R35 and the deletion mutants derived from it could be identified as a cluster, with *m134* showing more variation from the others on the basis of this reproductive gene set than found between R35 and *m115* (Fig. 2).

We then further examined the gene list for stage- or genotype-specific patterns using hierarchical clustering of a subset of 105 genes displaying variation exceeding 1 standard deviation of the mean. This revealed multiple patterns, including differential expression between stages that were shared across all genotypes, and also strong genotype-specific changes irrespective of stage (Additional file 2: Figure S2). Notably, however, neither the PCA nor the hierarchical cluster analyses of this reproductive gene set identified any discriminatory expression signatures between the sexual and apomictic phenotypes. However, the PCA does suggest that comparisons between the two apomictic species, R35 and D36, and in particular comparisons between genotypes within the R35 background, *m115* and *m134*, were likely to be the most informative with respect to common and differential gene expression changes during these developmental stages.

### Identification of additional *LOA*-linked markers

**Fig. 3:**
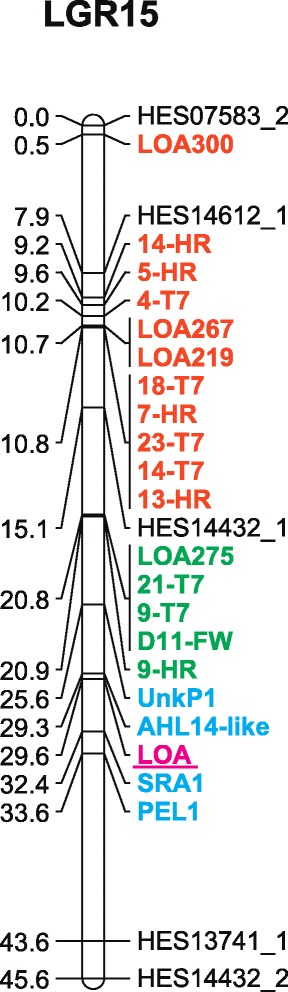
Linkage group 15 (*LGR15*) from R35 containing the *LOA* locus and linked markers. The linkage group, updated here, is part of a linkage map previously constructed in R35 [20]. The LOA trait is shown in *magenta type* and *underlined. LOA*-linked markers within both the *m134* and *m115* deletions are in *cyan type. LOA-*linked markers within the *m115* deletion are shown in *orange* and *green type* which represent their location upon previously defined *LOA-*linked contigs A and B, respectively [19]. Centimorgan distances are indicated at *left*

Previous analyses of *LOA-* and *LOP*-linked markers indicated that genomic deletions at the *LOA* and *LOP* loci in *m115* were extensive, whereas in *m134*, all *LOA-* and *LOP*-linked markers were found to be present [17]. This suggested that either the *LOA*-associated deletion within *m134* was smaller than that in *m115*, or alternatively, the loss of apospory phenotype in *m134* could be due to an *LOA*-independent lesion potentially acting downstream of *LOA*. To identify potential genes and markers linked to deletions in common between *m115* and *m134*, the ovary transcriptomes of R35 and the two deletion mutants were used to identify SNPs lost within both mutants. A total of 1054 and 957 transcriptome contigs from the MMC and FM stages, respectively, were identified as missing SNPs from both *m115* and *m134* mutant transcriptomes. A subset of 13 SNPs was then selected for validation and examination of linkage to *LOA* through the design of sequence characterized amplified region (SCAR) markers and amplification from a collection of *LOA* and *LOP* deletion mutants, as well as a previously described *Hieracium* mapping population [20]. The validation process confirmed that 4 of these markers are missing from all *LOA* deletion mutants tested, including *m134*, and that these genes are linked to *LOA* (Fig. 3; Additional file 1: Table S3). All but one of the 4 markers were also missing within the sexual P36 species, and in all but one recombinant all 4 markers were missing (Additional file 1: Table S3). These are the most closely linked markers to *LOA* identified to date, and demonstrate the utility of the transcriptomic resources for mapping purposes. Importantly, this work also demonstrates that *m134* contains a deletion genetically linked to the *LOA* locus, confirming its utility for use in examining transcriptional changes related to *LOA* function.

### Identification of small RNA pathway genes within *Hieracium* ovaries

Previous studies have shown that mutations affecting small RNA pathways in maize and *Arabidopsis* can exhibit defects in specification of sexual cell lineages or induce formation of extra cells with gametogenic potential [26, 27, 29, 39].We therefore interrogated the D18 genomic resource for predicted genes encoding members of the RDR, DCL and AGO protein families. Putative candidate genes were identified using blastp, and predicted *Hieracium* proteins were assigned names based on sequence similarity to annotated proteins from tomato [40, 41]. We identified 24 predicted genes encoding partial or full-length RDR-related proteins, 20 encoding predicted DCL proteins and 45 encoding predicted AGO proteins (Additional file 1: Table S4).

**Fig. 4:**
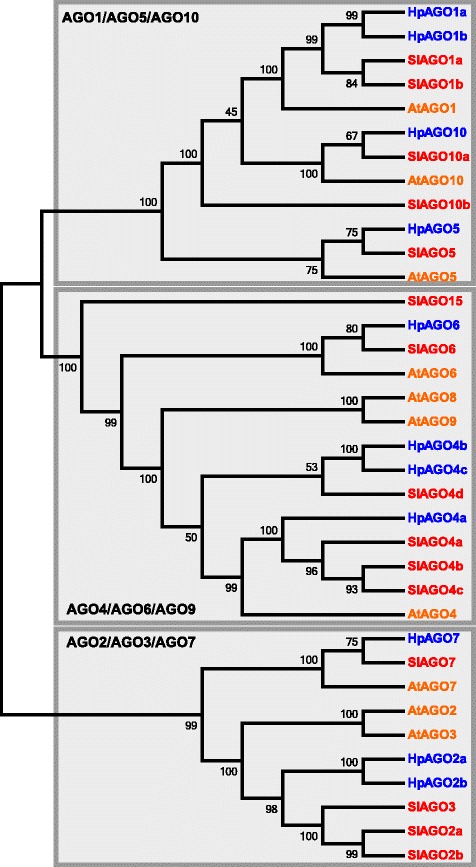
Phylogenetic analysis of cloned ARGONAUTE proteins from *Hieracium.* Consensus sequences from cloned R35 *Hieracium* AGO proteins (*HpAGO, blue*) were aligned to AGO proteins from *Solanum lycopersicum* (*SlAGO, red*) and *Arabidopsis thaliana* (*AtAGO, orange*). A cladogram was generated based on 1000 alignments. Bootstrap values are indicated on *branches. Hieracium* AGOs fell into three primary clades alongside orthologs in tomato and *Arabidopsis*

In addition to D18 genomic sequences, we compared expressed *AGO* sequences isolated from apomictic R35 and sexual P36 ovaries to investigate whether structural differences or gene family composition in ovary-expressed AGO proteins might contribute to their reproductive phenotypes. We identified 11 transcriptome contigs encoding AGO-related proteins with high similarity to tomato AGO proteins and confirmed the expression of the putative *AGO* genes by sequencing their predicted full-length coding regions from R35 and P36 whole ovary cDNA (Additional file 4). In most cases, the *AGO* reverse transcription-PCR (RT-PCR) products were heterogeneous and exhibited SNPs present in the transcriptome contigs as well as SNPs unique to the amplified products, suggesting that both the clones and assembled transcriptome contigs likely represent multiple gene copies of high sequence similarity (Additional file 4). A phylogenetic analysis of consensus sequences from all amplified *AGO*s placed the 11 *Hieracium* AGO proteins into three major clades with representative members of each typical plant AGO protein family (Fig. 4), suggesting small RNA pathways observed in other plant species are also likely to be functional within *Hieracium* ovaries. Direct orthologs of AtAGO8 or AtAGO9 were not identified within *Hieracium* or tomato in this analysis, despite playing an important role in restricting MMC specification within *Arabidopsis* [26]. Furthermore, AtAGO2a, AtAGO3, HpAGO2a, HpAGO2b, SlAGO2a, SlAGO2b and SlAGO3 appear to be paralogous within each respective species, suggesting that they arose from duplication following speciation.

**Fig. 5:**
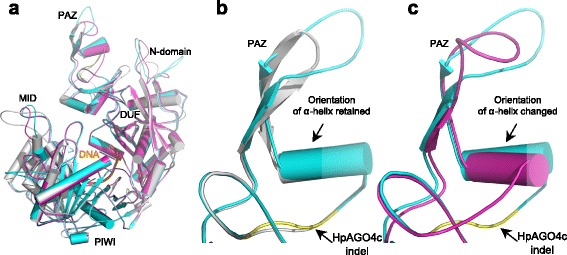
Protein modelling of HpAGO4c. **a** A structural super-position of the human Argonaute2 protein (*grey*) with HpAGO4cP36 (*cyan*) and HpAGO4cR35 (*magenta*). **b** A portion of the structural super-position of the human Argonaute2 protein (*grey*) with HpAGO4cP36 (*cyan*) showing the location of the HpAGO4cR35 deletion (*yellow*). The HpAGO4cP36 overlaps well with the human Argonaute2 protein. **c** A portion of the structural super-position of the HpAGO4cP36 (*cyan*) and HpAGO4cR35 (*magenta*) showing the location of the deletion in HpAGO4cR35 (*yellow*). The nine-amino-acid residue deletion in HpAGO4cR35 is predicted to shorten the secondary loop structure, which causes a substantial tilt of a neighbouring α-helix

Sequence comparisons of orthologous AGOs between sexual P36 and apomictic R35 plants showed high levels of sequence conservation with most orthologs sharing greater than 90 % pairwise sequence identity at the amino acid level (Additional file 4). Within the PAZ domain of the apomictic R35 HpAGO4c we identified a nine-amino-acid residue deletion (EYSADYPCL) that was not detected in clones from sexual P36. The deletion in R35 removes several conserved residues including a highly conserved proline (P357 in P36 HpAGO4c). The *Arabidopsis ago1-42* mutant is affected at the same residue (P481T) and has a strong phenotype most likely due to improper folding of the PAZ domain [42]. It is therefore likely that the R35 HpAGO4c protein is similarly severely affected in function. Modelling of the R35 HpAGO4c protein against the human Argonaute2 protein suggests that the deletion could cause a shift in the a7 α-helix (Fig. 5) [43], although it is unclear whether such a deletion would allow proper folding of the PAZ domain. We also identified a three-amino-acid residue indel within the PIWI domain of HpAGO7 between R35 and P36. The indel is in an evolutionarily highly variable region and is unlikely to affect the overall structure. Markers generated against the *HpAGO4c* and *HpAGO7* indels indicated they were not missing in either *m115* or *m134.* Therefore, these indels appear to be species-specific differences between R35 and P36, and are unlikely to be associated with the *LOA* locus (Additional file 1: Table S3).

We further examined all cloned *AGO* sequences for differential gene expression within ovaries using quantitative real-time PCR (qRT-PCR). We restricted this analysis to R35, *m115* and *m134* genotypes, as differences in gene expression can vary between species. We detected decreased expression of *HpAGO1a* within *m115* ovaries relative to R35 ovaries at both stages (Table 2), whereas *HpAGO2b* was downregulated in both *m115* and *m134* ovaries relative to R35 at both stages (Table 2). *HpAGO5* also appears to be slightly downregulated in both *m115* and *m134* ovaries relative to R35; however, only the R35:*m134* comparisons met statistical significance (Table 2). Further examination of SNPs within the ovary transcriptome suggested that three polymorphic SNPs from *HpAGO1a* were missing in the *m115* ovary transcriptome, suggesting that the reduced expression of *HpAGO1a* observed in *m115* ovaries might be due to the loss of one or more copies of *HpAGO1a* within this mutant. We confirmed the loss of these *HpAGO1a* polymorphic SNPs by amplifying them from genomic DNA and sequencing the resultant products. All three amplicons showed loss of the informative SNPs within *m115* and several other *LOA* deletion mutants, but not *m134*, suggesting they are not associated with the apospory phenotype (Additional file 1: Table S3).

**Table 2:** Relative expression of *HpAGO* genes in R35 ovaries relative to *m115* and *m134* ovaries as determined by qRT-PCR

HpAGO	R35:*m115* MMC AvglogFC (SD)	R35:*m115* FM AvglogFC (SD)	R35:*m134* MMC AvglogFC (SD)	R35:*m134* FM AvglogFC (SD)
*HpAGO1a*	0.71 (0.19)^a^	0.79 (0.16)^a^	−0.14 (0.38)	0.08 (0.11)
*HpAGO1b*	−0.67 (0.03)	−0.54 (0.04)	−0.6 (0.45)	−0.33 (0.14)
*HpAGO2a*	−0.26 (0.24)	0.05 (0.27)	−0.03 (0.17)	0.2 (0.4)
*HpAGO2b*	1.20 (0.71)^a^	1.12 (0.56)^a^	1.95 (0.07)^a^	1.55 (0.61)^a^
*HpAGO4a*	−0.37 (0.06)	0.12 (0.04)	−0.37 (0.18)	−0.32 (0.07)
*HpAGO4b*	−0.5 (0.04)	−0.44 (0.21)	−0.27 (0.09)	0.49 (0.21)
*HpAGO4c*	0.05 (0.27)	−0.04 (0.05)	0.27 (0.24)	0.51 (0.31)
*HpAGO5*	0.58 (0.4)	0.61 (0.45)	0.72 (0.41)^a^	0.68 (0.31)^a^
*HpAGO6*	−0.14 (0.12)	−0.03 (0.07)	−0.21 (0.23)	−0.08 (0.27)
*HpAGO7*	−0.16 (0.16)	−0.06 (0.08)	−0.73 (0.2)^a^	−0.27 (0.3)
*HpAGO10*	−0.01 (0.21)	−0.05 (0.07)	0 (0.3)	−0.17 (0.06)

*AvglogFC* average log_2_ fold change (ΔΔCt) of two biological replicates with two technical replicates, *SD* standard deviation

^a^Unpaired *t*test *P* value < 0.05

**Fig. 6:**
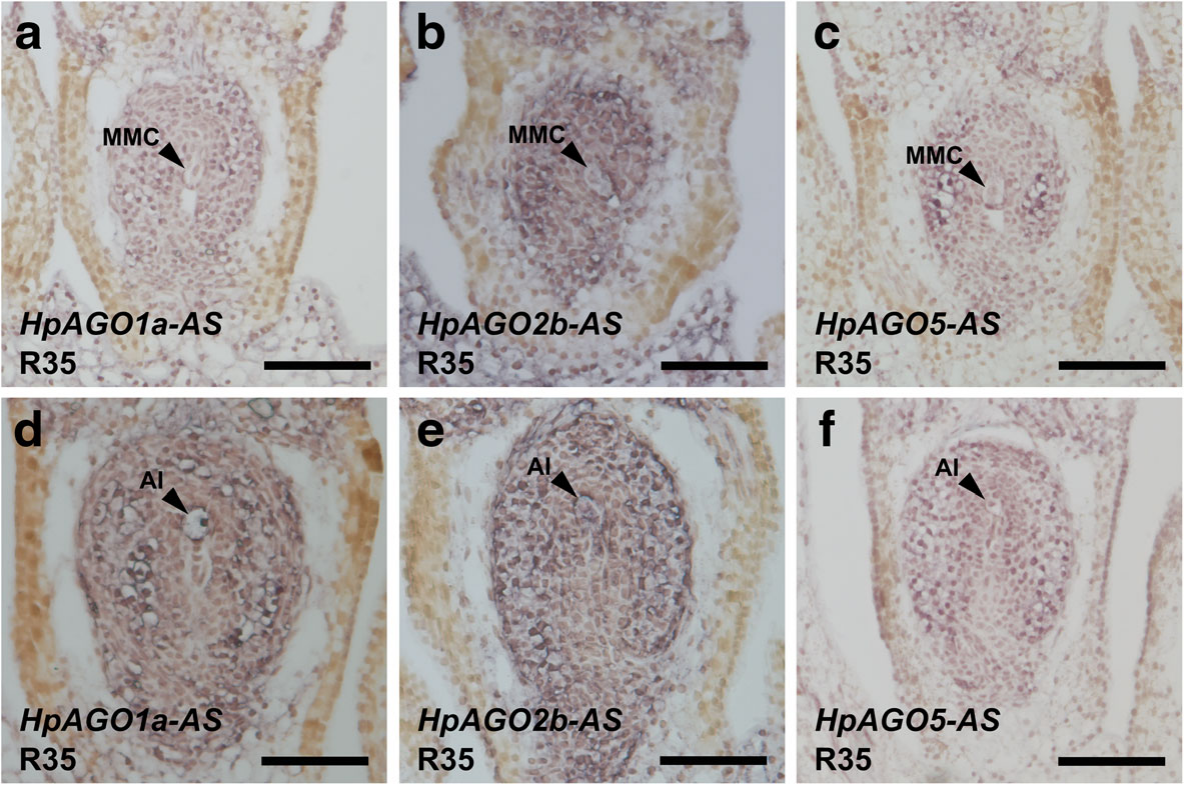
In situ hybridization of *HpAGO1a*, *HpAGO2b* and *HpAGO5* in *Hieracium*. **a** and **d** Hybridization of *HpAGO1a* antisense probe (*HpAGO1a-AS*) to MMC (**a**) and FM (**d**) stage ovary sections from R35. **b** and **e** Hybridization of *HpAGO2b* antisense probe (*HpAGO2b-AS*) to MMC (**b**) and FM (**e**) stage ovary sections from R35. **c** and **f** Hybridization of *HpAGO5* antisense probe (*HpAGO5-AS*) to MMC (**c**) and FM (**f**) stage ovary sections from R35. *MMC* megaspore mother cell, *AI* aposporous initial cell. Scale bars = 100 μm

As the qRT-PCR analysis suggested that *HpAGO1a*, *HpAGO2b* and *HpAGO5* were differentially expressed between R35 and the two derived deletion mutants, we further examined the expression of these genes in ovaries using in situ hybridization. In general, antisense probes against all three *AGO* genes showed broad expression patterns throughout the ovary and flower at both the MMC and FM stages (Fig. 6; Additional file 2: Figures S3, S4). Signals throughout the entire ovary were more intense at the FM stages compared to the MMC stages for all three *AGO* probes examined; however, there were no obvious spatial differences in signals between R35 samples and *m115* or *m134* (Additional file 2: Figures S3, S4). Although we could not reliably detect major differences in signal intensity between R35, *m115* or *m134* samples, this is most likely due to the relatively minor differential expression observed being below the level of detection using this method.

### Differential gene expression within aposporous ovaries during AI cell development

We next examined the ovary transcriptomic resource for differential gene expression between MMC and FM stages to identify genes associated with AI cell growth and development. Reads from apomictic R35 and D36 ovaries were aligned to D18 genomic contigs and predicted gene models. Within R35, 434 gene models displayed differences between the MMC and FM stages, and 179 (41.2 %) of these were also differential between MMC and FM stages in the D36 apomict (≥2 fold change, *P* ≤ 0.01 adjusted for multiple testing correction) (Additional file 1: Table S5, Additional file 3). This analysis identified a putative ortholog of the lipoxygenase *LOX2* (AT3G45140) as increasing in transcript abundance from the MMC to FM stage in R35, which corresponds with its increased expression during AI cell development as previously verified by in situ hybridization [9]. The 179 gene models commonly differential between both stages in R35 and D36 were mapped to GO molecular function and biological process terms through putative *Arabidopsis* orthologs. Enrichment of functional themes in differential lists was identified relative to a background of the full set of gene models found expressed in the R35 ovary at these stages. This analysis identified enrichments at the MMC stage for terms associated with DNA binding and transcription factors, as well as other pathways including polysaccharide biosynthesis and fatty acid metabolism (Additional file 1: Table S6). The FM stage lacked enrichment of transcription factor terms but was characterized by terms associated with proteolysis, cation transportand also arabinan and xylan catabolism (Additional file 1: Table S6). These results demonstrate our ability to detect AI-associated transcripts within our whole ovary transcriptomes and provide evidence of transcriptional changes during the initiation of AI cell development.

### *m115* and *m134* ovaries share many co-differentially expressed genes with contrasting directional fold changes

We further examined our transcriptome data to identify differentially expressed genes between ovaries from aposporous plants and those that lack the apospory phenotype. As our PCA demonstrated that genotype contributed the greatest amount of variation between samples (Fig. 2), we focussed our analysis on R35 and the two deletion mutants derived from it. Within the R35:*m115* comparisons, 6349 and 6896 predicted genes were identified as being differentially expressed at the MMC and FM stages, respectively (Additional file 3). Within the R35:*m134* comparisons, 5473 and 6374 predicted genes were identified as being differentially expressed at the MMC and FM stages, respectively (Additional file 3). We further examined 22 predicted differentially expressed genes using qRT-PCR. We were able to confirm significant differential expression in 10 of the 22 genes tested within cDNA isolated from whole ovaries (Table 3). The low validation rate may relate to complexities associated with cross-species comparisons between D18 predicted gene models and R35 transcripts, and/or the presence within the tetraploid plants of highly similar orthologs or homeologs with dissimilar expression profiles.

**Table 3:** Relative expression of putative differentially expressed predicted gene models in R35 ovaries relative to *m115* and *m134* ovaries as determined by qRT-PCR

Predicted gene ID	Annotation	R35:*m115* MMC AvglogFC (SD)	R35:*m115* FM AvglogFC (SD)	R35:*m134* MMC AvglogFC (SD)	R35:*m134* FM AvglogFC (SD)
augustus-D18g-s1064104.g619280	SDE3-related	nd	0.02 (0.13)	nd	0.34 (0.24)
augustus-D18g-s14661.g22078	Aluminium-induced protein	nd	0.34 (0.13)	nd	nd
augustus-D18g-s179972.g197694	EXORDIUM-related (*HpEXO-like*)	8.15 (0.53)^a^	6.00 (0.61)^a^	7.19 (1.85)^a^	4.66 (0.47)^a^
augustus-D18g-s204206.g212029	DUF584	nd	nd	nd	0.33 (0.11)
augustus-D18g-s221502.g225572	Glucan synthase-like	nd	0.04 (0.08)	nd	0.01 (0.08)
augustus-D18g-s227925.g234365	Cytochrome P450	nd	nd	5.79 (0.98)^a^	4.74 (1.33)^a^
augustus-D18g-s228894.g235636	S/T protein kinase	nd	−0.20 (0.35)	nd	−0.41 (0.12)
augustus-D18g-s250182.g259844	NRPD1B-related	nd	0.00 (0.14)	nd	0.66 (0.06)
augustus-D18g-s255108.g264963	Lipoxygenase (*HpLOX2*)	2.87 (0.37)^a^	2.40 (0.42)^a^	2.75 (1.92)^a^	1.94 (0.59)^a^
augustus-D18g-s276058.g281382	Nuclear matrix protein	nd	nd	nd	−0.03 (0.15)
augustus-D18g-s335972.g331650	Laccase	5.10 (0.30)^a^	2.84 (0.22)^a^	4.97 (0.50)^a^	4.06 (0.34)^a^
augustus-D18g-s33935.g43705	DCL4-related	nd	−0.04 (0.13)	nd	−0.12 (0.13)
augustus-D18g-s341112.g335498	LOB domain	1.83 (0.16)^a^	1.68 (0.23)^a^	1.99 (0.47)^a^	2.40 (0.10)^a^
augustus-D18g-s541698.g482507	Pentatricopeptide	−2.64 (0.28)^a^	−3.08 (0.21)^a^	−2.52 (0.45)^a^	−2.69 (0.09)^a^
augustus-D18g-s562621.g490922	DCL2-related	nd	−0.19 (0.46)	nd	0.15 (0.09)
augustus-D18g-s569078.g495478	CBS domain	nd	nd	nd	0.38 (0.08)
augustus-D18g-s573721.g500128	TIR-2 TMV R	19.61 (0.05)^a^	15.73 (3.55)^a^	19.74 (0.42)^a^	18.34 (1.83)^a^
augustus-D18g-s636193.g536033	Cinnamic acid-dihydrodiol	nd	−0.14 (1.6)	nd	nd
augustus-D18g-s724133.g556087	Alcohol dehydrogenase	1.87 (0.56)^a^	1.94 (0.41)^a^	nd	nd
augustus-D18g-s742950.g560669	Myrosinase binding	4.45 (0.75)^a^	5.44 (0.77)^a^	nd	nd
augustus-D18g-s766362.g567064	Metal ion binding protein	nd	0.28 (0.16)	nd	nd
augustus-D18g-s857898.g586359	DUF674	nd	nd	5.04 (0.46)^a^	5.11 (0.08)^a^

*AvglogFC* average log_2_ fold change (ΔΔCt) of two biological replicates with two technical replicates

*SD* standard deviation

*nd* not determined

^a^Unpaired *t*test *P* value < 0.05

**Fig. 7:**
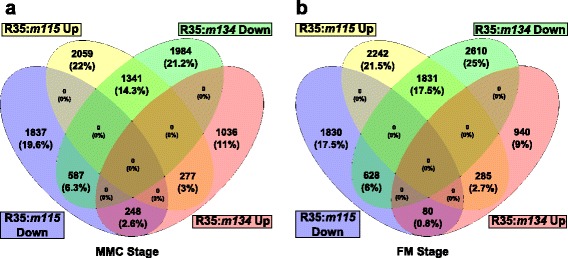
Overlapping differentially expressed genes between R35 and mutant apomict comparisons at two developmental stages. **a** A four-way Venn diagram showing numbers of overlapping differentially expressed genes between the R35:*m115* and R35:*m134* comparisons at the MMC stage. **b** A four-way Venn diagram showing numbers of overlapping differentially expressed genes between the R35:*m115* and R35:*m134* comparisons at the FM stage

We then examined whether there were any overlaps within the differentially expressed predicted genes between the R35:*m115* and R35:*m134* comparisons. We identified 2453 and 2824 predicted gene models with differential gene expression in both mutants at the MMC and FM stages, respectively (Fig. 7; Additional file 3). We found that the AI cell-associated *HpLOX2* gene was downregulated in both *m115* and *m134* mutants, which is consistent with the sexual phenotype of these plants. Of the differentially expressed genes in common between *m115* and *m134*, 277 at the MMC stage and 285 at the FM stage showed decreased expression in both mutants relative to R35 (Fig. 7; Additional file 3). However, at both the MMC and FM stages, the largest overlaps of differentially expressed predicted genes between the R35:*m115* and R35:*m134* comparisons were associated with opposing expression patterns, having increased expression in *m134* ovaries and decreased expression in *m115* ovaries relative to R35 (Fig. 7; Additional file 3).

We further examined differentially expressed genes in common between both R35:*m115* and R35:*m134* comparisons for functional themes within enriched GO terms. Due to the low annotation rate within differentially expressed genes showing similar directionality between the R35:*m115* and R35:*m134* comparisons (Additional file 3), we instead focussed on enriched GO terms within all differentially expressed genes in common, irrespective of directionality. Differentially expressed genes at both stages were enriched for terms involved in chromatin silencing and small RNA production, including maintenance of DNA methylation, gene silencing by RNA, post-transcriptional gene silencing by RNA and production of siRNA involved in RNA interference (Additional file 3). Genes that fell within these categories include genes annotated as putative orthologs of *Arabidopsis* shown to be involved in the biosynthesis of small RNAs, including *RDR2*, *DCL2*, *DCL3* and *DCL4*, as well as other associated genes such as *DECREASE IN DNA METHYLATION1* (*DDM1*), *SILENCING DEFECTIVE3* (*SDE3*), subunits of RNA polymerase IV (*NRPD1A*) and RNA polymerase V (*NRPD1B*), *AGO4* and *AGO10*. Interestingly, in *RDR2* and *DCL3* mutants, as well as the *nrpd1a nrpd1b* double mutant, additional MMC-like cells are specified in *Arabidopsis* [26]. As with most differentially expressed genes in our transcriptome analysis, these genes showed decreased expression in *m115* ovaries and increased expression in *m134* ovaries relative to R35. We attempted to confirm the differential expression of putative orthologs of *SDE3*, *DCL2*, *DCL4* and *NRPD1B* using qRT-PCR; however, we were unable to detect differential expression using this method (Table 3). Other enriched GO terms in both stages included terms associated with regulation of cell cycle, chromosome segregation during mitosis and meiosis, stem cell fate, cell wall biosynthesis and regulation of cell growth (Additional file 3).

### Apomixis initiation is dependent on localized signals

Certain classes of small RNAs and mRNAs are involved in long distance movement from roots and vegetative tissues to influence various processes [44–47]. The differential and divergent expression of genes involved in small RNA pathways identified in comparisons between R35 and the aposporous mutants *m115* and *m134* prompted us to examine if long distance signalling influenced apomixis initiation. We conducted reciprocal grafting experiments combining scions and rootstocks of sexual and apomictic seedlings to test if signals from the roots of apomictic plants could induce AI cells in ovules formed in flowers of sexual scions, or if signals from sexual rootstocks could suppress AI cell formation in ovules of an apomict scion (Table 4). AI-like cells were absent at flowering in ovules of control grafts combining sexual P36 scions and rootstocks. Al cells were only found within ovules when the grafted scion was the R35 apomict, and suppression of apomixis was not observed when the sexual seedling was used as the rootstock (Table 4). This shows that apomixis is not graft transmissible and suggests that the major signals required to induce AI cell development are likely to be localized within floral and ovary tissues and unlikely to occur via long distance signalling.

**Table 4:** Percent of ovules showing evidence of AI cell development in grafted plants

Scion/rootstock	% AI cells formed	% Aborted embryo sacs	Number of plants (ovules scored)
P36/P36	0 %	28 %	5 (536)
R35/R35	51 %	37 %	5 (487)
R35/P36	56 %	35 %	7 (681)
P36/R35	<1 %	62 %	7 (669)

### Analysis of small RNA profiles within sexual and apomictic ovaries and their targets

To directly characterize the composition of small RNAs expressed within *Hieracium* ovaries, we sequenced small RNAs isolated from whole ovaries at both the MMC and FM stages within the aposporous apomicts R35 and D36, as well as deletion mutants *m115* and *m134*. As a comparison, small RNAs were also sequenced from the leaves of each genotype. In total, the filtered dataset contained 376.4 million sequences (10–30 nt) (Additional file 1: Table S1). Length distributions were used to assess whether the sequence diversity and abundance relationship was qualitatively similar across *Hieracium* samples and whether global length and diversity patterns reflected those reported in other plant tissues (Additional file 5) [48, 49]. Length distributions of total small RNA counts across all samples revealed a doubling of 22-nt small RNAs in the ovary samples relative to leaf samples in all genotypes (Additional file 5). The increase in total 22-nt small RNAs in ovaries relative to leaves may represent an organ-specific signature. Length distributions of small RNAs within R35, *m115* and *m134* ovaries suggested a small decrease in the number of 21–22 nt small RNAs and a small increase in 24-nt small RNAs within both mutants (Additional file 2: Figure S5, Additional file 5). However, we did not detect any major differences in small RNA compositions between R35, *m115* or *m134* ovaries at either developmental stage.

We aligned the full set of small RNA sequences to the assembled D18 genomic contigs. On average we were able to align 91.1 % of small RNA sequences from ovary and leaf samples to D18 contigs, of which 52.3 % aligned to genic regions (Additional file 1: Table S1, Additional file 5). Genic regions were defined by the coordinates of *in silico* predicted gene models extended 500 bp upstream and downstream to cover potential regulatory regions. On average 62 % of small RNAs from R35 ovaries aligned to predicted gene bodies (CDSs and introns), 19 % aligned to predicted untranslated regions (UTRs) and 19 % aligned to +/−500 bp upstream/downstream regions (Additional file 5). In comparisons of alignments between 21-nt and 24-nt small RNAs, 21-nt small RNAs were more likely to align to predicted CDSs and 5’ UTRs, whereas 24-nt small RNAs aligned more frequently to predicted 3’ UTRs and downstream regions (Additional file 5). Alignment distributions within predicted gene models across all samples analysed were highly similar, and we did not observe any differences between R35, *m115* and *m134* at either stage (Additional file 5).

Small RNA sequences were also used to predict and annotate microRNAs (miRNA). Using the small RNA sequence directly, we could identify 266 distinct matches to previously reported plant miRNAs present in miRBase [50], and of these 162 could be aligned to locations in the D18 assembly. To predict potentially novel miRNAs in the D18 genomic assembly, we looked for patterns of small RNA alignment and a predicted secondary structure suggestive of an miRNA-precursor-like hairpin. We identified 352 predicted *MIRNA* genes within the D18 assembly, of which 81 could be annotated using the miRBase database, suggesting the majority of putative *MIRNA* genes are likely species-specific (Additional files 5 and 6). In total, 69 miRNA annotatable small RNA reads and 48 predicted miRNAs were found to have differential small RNA read counts in comparisons between R35 and *m115* or *m134* (Additional file 5). The list of differentially expressed miRNAs included annotations to *miR169*, *miR397*, *miR396*, *miR408*, *miR399*, *miR167*, *miR393*, *miR390*, *miR156* and *miR171*; however, we did not observe any conserved pattern of differential expression between sexual and apomictic ovaries (Additional file 5).

We further investigated genic regions for evidence of differential accumulation of small RNAs between R35, *m115* and *m134* at both the MMC and FM stages. Within the R35:*m115* comparisons, 10,357 and 3065 predicted gene models showed greater than twofold differential accumulation of small RNA alignments at the MMC and FM stages, respectively (Additional file 5). Within the R35:*m134* comparisons, 5850 and 3370 predicted gene models exhibited differential accumulation of small RNA alignments at the MMC and FM stages, respectively (Additional file 5). The differential gene models in common between the small RNA mutant comparisons were 1309 and 1319 at the MMC and FM stages, respectively (Additional file 5).

We further examined whether any of the predicted genes with differential small RNA counts overlapped with predicted differential expression in our transcriptome analysis. Predicted gene models that had complementary fold change directions between the transcriptome and small RNA alignments were examined to identify potential genes in which the changes in small RNA accumulation might be affecting transcript abundance (Additional file 5). Cross-referencing genes that appeared across multiple comparisons identified 40 genes that had complementary differential expression in two or more lists (Additional file 1: Table S7). In examining annotated genes within these lists, we identified two gene models annotated as *EXORDIUM-like* (*HpEXO-like*) genes [51], both of which had decreased transcript abundance that correlated with an increase of 24-nt small RNAs in both mutants relative to R35 (Additional file 1: Table S7). Both gene models predominantly showed an accumulation of 24-nt small RNAs within the gene body, suggesting these two gene models might be targets of the RdDM transcriptional gene silencing pathway (Additional file 2: Figure S6). The two *HpEXO-like* genes are highly similar in sequence, sharing 98 % identity at both the nucleotide and predicted amino acid sequence level. As such, the majority of differentially abundant small RNAs are predicted to target both gene models at locations with identical sequence. Due to high sequence similarity between these two gene models, we were unable to design oligos that could distinguish between the two *HpEXO-like* isoforms using qRT-PCR. However, a pair of oligos which amplifies both *HpEXO-like* isoforms did confirm a significant downregulation of transcript abundance within both mutants (Table 3). We were able to amplify the *HpEXO-like* targets from genomic DNA isolated from both *m115* and *m134*, suggesting downregulation of these *HpEXO-like* genes within mutant ovaries is most likely due to transcriptional repression rather than deletion. However, it is also possible that an identical *HpEXO-like* gene has been deleted in both mutants.

## Discussion

### Integrating the *Hieracium* genomic and transcriptomic resources to identify differentially expressed genes

Previous studies within apomictic species have focussed on transcriptomic approaches. While a few candidate genes have been identified in these studies [6, 8–10], the analysis of transcriptomes within most apomictic species is complicated by the high complexity, redundancy and polyploid nature of their genomes. The limitations of de novo transcriptomic assemblies in the absence of genomic information frequently leads to the compression of multiple gene copies into single transcriptome contigs, effectively reducing the gene space, and potential loss of informative alleles [11, 12]. By taking advantage of the dihaploid D18 apomictic plant, we have maximized the genomic sequencing coverage of a functional apomict, which would be highly unfeasible using a typically tetraploid genotype.

Using our D18 genomic assembly as a tool for transcriptomic analysis, we compared the transcriptomes of whole ovaries from both sexual and apomictic plants. The ovaries were isolated at two stages, MMC and FM, which respectively cover early meiotic phases and early female gametophyte development within the ovules of all genotypes examined in this study. As the transcriptomes were generated from whole ovaries, which contain both gametophytic and sporophytic tissue, in addition to a small amount of aposporous tissue in apomicts, it is important to recognize the limitations of using such starting tissue for the detection of apospory tissue-specific gene expression. Nevertheless, we were able to detect differential expression of the AI-associated gene *HpLOX2* (Table 3 and Additional file 3), suggesting that this approach is not impractical. This strategy also provides an opportunity to capture transcript information relating to sporophytic ovule signals, as these may also contribute to gametogenic cell specification and gametophyte development. Our whole ovary transcriptomic and genomic resources will prove invaluable for future research using laser-assisted microdissection approaches to analyse tissue-specific expression, by improving our ability to assemble contigs and distinguish unique transcripts, which is often a difficult task when using these approaches.

Confidence in the ability to detect low abundance transcripts was supported by the analysis of all transcriptomes using a subset of orthologs associated with the sexual pathways in *Arabidopsis,* which demonstrated detection of genes expected to be expressed at low abundance in the ovary (Additional file 3). These analyses also demonstrated that genotype differences were a major contributor to variation in sexual gene expression, stressing the need to explore the use of closely related species, segregants and/or mutants within the same background for meaningful transcriptomic comparison (Fig. 2). Importantly, these analyses also confirmed findings from our previous work in *Hieracium* that has found that the sexual pathway remains viable in apomictic ovary samples, supporting the concept that similar gene expression pathways are recruited in sexual and apomictic ovaries [9, 52].

While the assemblies derived cannot be considered to provide full coverage of the D18 genome or ovary transcriptomes, their size, internal consistency and degree of overlap within the resource and to related genomic resources is high (Additional file 1: Table S1), suggesting excellent coverage of the early reproductive developmental transcriptome within *Hieracium* ovaries. To our knowledge, it is the first publically available genomic resource available within any apomictic species, integrating transcriptomic and small RNA sequencing data from multiple genotypes. Thus, this collection of sequences is established as a coherent and targeted functional genomic resource for the study of gene expression programs occurring during the early sexual and apomictic events of gametogenesis.

### The extensive nature of *Hieracium RDR*, *DCL* and *AGO* gene families

Small RNA pathways have been implicated in the regulation of sexual pathway initiation in other species [26, 27, 29, 39], therefore, we initiated a search for *RDR*, *DCL* and *AGO* genes within the D18 genomic assembly. This revealed a larger than expected number of predicted genes within these families, especially when considering the incomplete coverage of the chromosomally reduced D18 genomic assembly. However, *RDR*, *DCL* and *AGO* family members involved in major small RNA biogenesis pathways were represented within the D18 genomic assembly, suggesting that a complete loss of any one pathway is unlikely to explain the apospory phenotype (Additional file 1: Table S4). Although we did not identify direct orthologs of *Arabidopsis AGO8* or *AGO9* in *Hieracium*, this is not unexpected, as phylogenetic analyses of *AGO*s in other plant species also demonstrate a lack of direct orthologs to these genes, suggesting that *AGO8* and *AGO9* are *Arabidopsis*-specific [40, 53–55]. Further examination of ovary-expressed *AGO* genes by cloning of full-length cDNAs from apomictic R35 and sexual P36 ovaries revealed that representatives of each *AGO* were expressed in both species. Although some differences between *AGO*s isolated from R35 and P36 do exist, they do not appear to be associated with the *LOA* locus and are most likely species-specific differences (Additional file 1: Table S3, Additional file 4).

qRT-PCR analysis of all cloned ovary-expressed *AGO* genes identified *HpAGO2b* and *HpAGO5* as having reduced expression in one or both mutants. *HpAGO2b* showed the strongest reduction in both *m115* and *m134* and was downregulated at both stages examined (Table 2). *HpAGO2b* is most closely related to *AtAGO2* and *AtAGO3*, although *HpAGO2a* and *HpAGO2b* form their own clade (Fig. 4); therefore, it is not possible to assign a direct ortholog. *AtAGO2* has been shown to preferentially bind small RNAs 21 nt in length, and has been implicated in multiple small RNA response pathways, including post-transcriptional gene silencing in response to viral or bacterial defence as well as DNA damage repair [56–59]. In contrast, *AtAGO3* has been demonstrated to primarily bind siRNAs 24 nt in length, and exhibits partial functional redundancy with *AtAGO4* in the RdDM pathway despite being more closely related to *AtAGO2* [60]. *HpAGO5* was most strongly downregulated in *m134* at both stages, and may also be downregulated in *m115*, although it failed to meet the statistical cut-off (Table 2). In *Arabidopsis*, *AtAGO5* has also been shown to preferentially bind small RNAs 21 nt in length, and is expressed in developing ovules and anthers [61]. A dominant mutation affecting *AtAGO5* inhibits megagametogenesis following specification of the MMC; however, plants with null mutations in *AtAGO5* are fertile [29]. Orthologs of *AtAGO5* such as *MEL1* in rice and *AGO5c* in maize are also expressed within developing anthers and ovules, and mutations affecting *MEL1* cause arrest of meiosis and male sterility [62, 63].

Whether *HpAGO2b* or *HpAGO5* function in similar small RNA pathways as their *Arabidopsis* orthologs within *Hieracium* ovaries has yet to be determined. Copies of *HpAGO2b* and *HpAGO5* do not appear to be missing from either the *m115* or *m134* genomes; however, their downregulation does suggest they could act downstream of processes required for the initiation of apospory. In situ analyses demonstrated that these two *AGO* genes are broadly expressed throughout the ovary and flower (Fig. 6; Additional file 2: Figures S3,S4), and further analysis using protein-specific antibodies could provide additional information regarding their function during AI cell development.

### The majority of differentially expressed genes in *m115* and *m134* ovaries exhibit opposing directionality

In analysing differential gene expression of whole ovaries during AI cell development, we focussed on the two deletion mutants, *m115* and *m134*, which are derived from the R35 background. By examining ovaries from plants within the same background, noise from cross-species differences could be reduced as much as possible. Additionally, *m115* and *m134* are part of a larger collection of deletion mutants in R35 [16, 18], and an existing mapping population within R35 provides a rapid means to determine linkage of identified candidate genes to the *LOA* locus [20].

Comparisons between ovaries from R35 and the two deletion mutants (*m115* and *m134*) showed that the deletion mutants shared a large fraction of differentially expressed predicted gene models as compared to R35 at both stages examined (Fig. 7; Additional file 3). Interestingly, although many predicted gene models were commonly differentially expressed within *m115* and *m134*, their fold changes were frequently found to be in opposing directions, with the majority of gene models showing increased expression in *m134* ovaries and decreased expression in *m115* ovaries relative to R35 (Fig. 7; Additional file 3). Differentially expressed gene models that fell into this category made up greater than half of all differentially expressed genes in common between the two mutant comparisons at both stages. This would suggest that although the two mutants are phenotypically similar at the stages examined, the phenotypes are possibly the result of different causal mutations that affect common downstream pathways, or alternatively, similar causal mutations that are also affected by additional mutations in downstream pathways. It is also possible that the large number of commonly differentially expressed genes may not be related to the phenotype at all, or could be the result of similar large-scale genomic differences.

One possible explanation could be the genomic constituencies of the plants examined. Gamma irradiation, through which *m115* and *m134* were generated, is known to cause large-scale genomic deletions and rearrangements, and occasionally affects chromosomal inheritance [19, 32, 33]. R35, the background plant from which both *m115* and *m134* were generated, is a known aneuploid (2*n* = 4x-1 = 35), whereas *m134* plants have an extra chromosome (2*n* = 4x = 36) (Additional file 2: Figure S1) [19]. The extra chromosome does not appear to carry the *LOA* locus (Additional file 2: Figure S1). The karyotype of *m115* is currently unknown; however, *m115* is thought to contain significant large-scale deletions, as most *LOA-* and *LOP*-linked markers examined to date have been found to be missing within this mutant [18]. Therefore, it is possible that differentially expressed gene models with opposing fold change directions might be explained by loss or duplication of a similar chromosome or region.

Differentially expressed predicted gene models in common between *m115* and *m134* were enriched for genes with GO categories associated with chromatin silencing, RNA-dependent DNA methylation and small RNA biogenesis (Additional file 3). Many of the genes found to be differentially expressed within these GO categories have been shown to affect sexual lineage specification with *Arabidopsis* and maize, including putative orthologs of *ZmAGO4d*, *AtRDR2*, *AtDCL3*, *AtDCL4* and subunits of RNA polymerases IV and V [26, 27]. All of these putative orthologs showed opposing fold change directions, with decreased expression in *m115* ovaries and increased expression in *m134* ovaries relative to R35 at both stages (Additional file 3). Therefore, it is difficult to conclude whether the observed perturbation of these pathways is directly associated with loss of the apospory phenotype in these mutants.

### Integration of small RNA and transcriptome data identifies putative siRNA targets

Our transcriptome analysis of R35, *m115* and *m134* ovaries suggested that a number of small RNA biogenesis genes were differentially expressed as compared to R35 ovaries. However, comparisons of total small RNA size distributions did not reveal any large-scale disruption in proportions of small RNA size classes (Additional file 2: Figure S5). This may be due to whole ovaries being used for this analysis, which would likely overwhelm any tissue-specific signals in small RNA distributions.

Despite this, a small number of genes differentially targeted by small RNAs in both *m115* and *m134* were identified (Additional file 5). An analysis of small RNA targets with corresponding differential gene expression identified two putative *EXO-like* genes to be downregulated in *m115* and *m134* ovaries, possibly through the RdDM pathway, as both show an increase of highly similar 24-nt small RNAs and decreased expression (Table 3, Additional file 1: Table S7 and Additional file 5). The function of *EXO-like* genes is not well characterized; however, they appear to be secreted into the cell wall, and are required for cell growth [51, 64–66]. *EXO-like* genes have also been reported to be required for brassinosteroid-induced signalling [51, 64–66]. However, targeting of these genes by RdDM in other species has not been previously reported. Upregulation of the *HpEXO-like* genes in R35 may represent growth and expansion of the AI cell, although further experiments will be required to determine if *HpEXO-like* genes are required for apospory.

### Potential candidate genes in AI cell specification

**Fig. 8:**
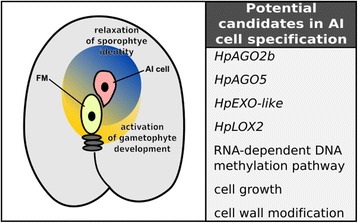
Summary of potential candidate genes involved in AI cell specification. A schematic of an ovule from aposporous *Hieracium* depicting postulated conditions required for the initiation of AI cell development. The relaxation of sporophyte identity (*blue shading*), in conjunction with the perception of signals to initiate gametophyte development (*orange shading*), leads to specification of AI cells with a mitotic gametophyte identity. Potential genes and pathways identified in this analysis that may play a role in leading to the postulated conditions required for AI cell specification are listed on the right

Specification of AI cell development within aposporous species of *Hieracium* is postulated to require two non-mutually exclusive conditions (Fig. 8). First, relaxation of sporophytic cell identity in the ovule tissue surrounding the sexually derived gametogenic cells seems probable. The second condition is the perception of signals for the specification and initiation of gametophyte development by those sporophytic cells, which is almost certain, as inhibiting meiosis in aposporous ovaries prevents AI cell initiation [18]. Whether or how the *LOA* locus is involved in either of these processes is currently poorly understood. Following AI cell specification, development of the aposporous gametophyte would undoubtedly share many of the regulatory pathways required for the mitotic events of sexual gametophyte development [52].

In the presentation of our newly generated genomic and transcriptomic resource, we performed an initial analysis of transcriptional changes within ovaries during the early stages of AI cell specification. In our analysis we identified a number of candidates that could potentially play a role in the specification and initiation of AI cells (Fig. 8). For example, *HpAGO2b*, *HpAGO5*, *HpEXO*-*like* and *HpLOX2* appear to be downregulated in ovaries from both *m115* and *m134* relative to R35. The function of these genes within *Hieracium* is currently unknown, but their orthologous functions in other species make them intriguing candidates. Other broader transcriptional changes in common between *m115* and *m134* ovaries, such as the perturbation of the RdDM pathway, or genes involved in cell growth and cell wall modifications may represent a loss of AI-permissive conditions within these mutant ovaries. But it is also quite possible that the observed changes could be unrelated to AI cell development.

With the exception of *HpLOX2*, which has been previously shown to be associated with the AI cell [9], the localization of differential expression within ovaries of these genes is unknown, and as whole ovary tissue was used in this initial analysis, it is difficult to place the observed changes into a structured regulatory pathway. Nevertheless, future research should clarify any roles these genes might play, and the resources generated here will play an invaluable role in these analyses.

## Conclusion

We have introduced a comprehensive toolkit for research into the early events of apomictic initiation observed in *Hieracium* species. The developed toolkit includes a previously unavailable genomic assembly coupled to transcriptome and small RNA sequencing of both sexual and apomictic ovaries at two developmental stages across early aposporous and sexual events. This toolkit will significantly support research towards uncovering the molecular events underpinning the apospory phenotype. We have demonstrated the efficacy of these developed resources by using them to identify common deleted genomic regions within *m115* and *m134* that are linked to *LOA*, in addition to comparing differences in small RNA pathways between ovaries of sexual and apomictic plants. Future work will focus on clarifying the role of these observed differences in sexual and apomictic *Hieracium* species, with particular emphasis on using additional transcriptomes generated through laser-assisted microdissection to identify cell type-specific genes for functional testing. The D18 genomic resource will also prove useful in the analysis of other aspects of research in *Hieracium,* such as autonomous seed formation.

## Methods

### Biological material

Six accessions of *Hieracium* subgenus *Pilosella* were employed in this study: *Hieracium piloselloides* (D36), *Hieracium praealtum* (R35), *Hieracium pilosella* (P36), *m115* and *m134*, which were derived from R35 [16] and D18 derived from D36 by parthenogenesis of a rare meiotically reduced egg [15, 18, 30]. The species have a base chromosome number of *n* = 9. The mean 2C values of genome size range from 7.03 pg in diploids to 16.67 in tetraploid accessions [67]. Apomictic species of *Hieracium* are facultative; the proportion of ovules forming AI cells and undergoing fertilization-independent seed formation within D36 and R35 is estimated to be ~97 % and ~99 %, respectively [15, 20]. Plants were vegetatively micropropagated to maintain clonal integrity. Plant growth conditions and AI phenotyping methods have been described previously [18, 24]. Grafting was performed as previously described [44, 68]. After flowering, ovaries formed in the grafted scion were fixed, cleared and scored at capitulum stages 4 and 10 for the presence or absence of enlarged AI-like cells in the ovule, defining the capability to initiate apomixis. Extent of embryo sac abortion was also scored. Chromosome spreads and fluorescence in situ hybridization (FISH) with an LOA267.14 BAC probe were performed as previously described [19].

### D18 genomic DNA sequencing and assembly

FISH experiments confirmed that D18 consists of 18 chromosomes including the *LOA*-carrying chromosome, together with conserved *LOA*-linked markers [17]. D18 genomic DNA was extracted from leaves using an adaption of the nuclear DNA enrichment method (Protocol C) described in [69]. Illumina sequencing of the D18 genomic DNA was undertaken by the Australian Genome Research Facility using a combination of 2 × 100 bp short insert (SI) paired-end and 2 × 100 bp standard insert paired-end sequencing using a HiSeq 2000 system.

The SI set generated fragment lengths distributed around 180 bp,therefore allowing 100 bp paired-end sequence reads from either end of the fragment to overlap. Prior to assembly,genomic reads were preprocessed to remove adapter- and/or vector-contaminated sequences, sequences containing N’s, excess exact duplicate read pairs and isolated sequences that did not substantially overlap another sequence in the dataset. SI reads were processed in an additional step to merge overlapping SI reads. Processed reads from DNA sequencing were assembled using the BioKanga assembly algorithm (https://sourceforge.net/projects/biokanga/).

The resulting genomic contigs were annotated for putative genic regions with the AUGUSTUS gene model prediction algorithm, with tomato as the training species, allowing for partial predictions and UTR predictions [70]. Both the genomic resource as a whole and translated predicted gene sequences were further annotated with alignments to known protein sequence sets from *Arabidopsis* (TAIR10), tomato (ITAG2.4), rice (MSU7), sorghum (Phytozome10 Sbicolor2.1), *Physcomitrella patens* (Phytozome10 PPatens3.0), *Zea mays* (Phytozome10 Zmays6a) and lettuce ESTs (National Center for Biotechnology Information, NCBI). Alignments to protein sequences were completed using blastp or tblastn as required, while genomic to EST alignments used a custom Blat-like algorithm, Blitz (https://sourceforge.net/projects/biokanga/). Blastp and tblastn alignments of predicted D18 protein sequences or CDSs to other species’ protein sequences reported up to 10 alignments for each D18 protein that met an e-value threshold of 1e-50 and had ≥50 % of D18 protein length covered by the alignment. For the purposes of the sexual pathway analysis, the top hit was chosen. Blitz alignments of EST sequences to D18 genomic sequences reported up to 10 alignments per EST sequence, with ≥60 % of EST sequence length included in alignment. Newly assembled *Hieracium* transcriptome contigs were also aligned to the D18 genomic contigs using Blitz with parameters as above. Sequence redundancy across the genomic contig set was assessed using a custom program to calculate edit (Hamming) distance between all possible 100-bp kmers within the assembly.

### Transcriptome and small RNA sequencing

To generate the transcriptomic resource, whole ovaries were dissected and collected into liquid nitrogen. Total RNA was isolated from ovary and leaf tissue and fractionated into mRNA (polyA) and small RNA (mirVana miRNA Isolation Kit, Life Technologies). Selection of small RNAs (<35 nt) with polyacrylamide gel electrophoresis was performed during library preparation. Illumina sequencing of the small RNA libraries using 50-bp single-end sequencing and sequencing of the mRNA libraries using 100-bp sequencing was performed by the Australian Genome Research Facility using a HiSeq 2000 system. Two biological replicates of mRNA and one replicate of small RNA were sequenced. Raw reads from both were trimmed for adapter and low quality sequence, and small RNA sequence sets were filtered to retain lengths of 18 to 25 nucleotides. Only sequences observed a minimum of five times (reads) in any one sample were further analysed. RNA sequencing libraries were preprocessed to remove adapter sequences and low quality ends using the sequence trimming algorithm (mcf) and assembled as single-ended reads using the Trinity transcriptome assembly pipeline [71].

### *m115* and *m134* SNP discovery

R35 ovule transcriptome contigs from both MMC and FM stages were used as a reference to realign RNA sequencing reads from the R35, *m115* and *m134* transcriptomes. Alignments were performed using the BioKanga aligner, allowing for reads to align to multiple loci and to have up to 10 % of the read length as substitutions. SNP discovery was based on two possible scenarios. The first scenario assumes that genes deleted from *m134* and *m115* contain a homoeologous or paralogous gene copy within the R35 genome. SNPs identified as heterozygous (AB) in R35 and homozygous (AA) in both *m115* and *m134* were selected for this category. The second scenario assumes hemizygosity of the deleted genes, and SNPs were therefore selected based on a homozygous genotype (A-) in R35 and an absence of reads (−−) aligned from both *m115* and *m134* mutants. SNP discovery was performed using BioKanga and required a minor allele frequency of at least 0.25 at any SNP locus. Sequences of *LOA*-linked contigs A and B [19] were used to identify syntenic regions within a genetic map of lettuce [22]. R35 transcriptome contigs identified from the SNP discovery process with similarity to lettuce ESTs within this syntenic region were considered for further analysis. To determine linkage to *LOA*, SCAR markers were designed and amplified from 9 *LOA* deletion mutants, 10 *LOP* deletion mutants and a subset of 42 progeny from a previously described R35 mapping population [20]. Primer sequences and PCR conditions are specified in Additional file 1: Table S8. Validated markers were mapped onto the R35 linkage map using JoinMap 4.0 as previously described [20].

### Differential expression and gene ontology analysis

To generate read counts for each predicted *Hieracium* gene model, RNA sequencing reads were aligned to the D18 genomic contig set using the BioKanga aligner, accepting only uniquely aligning reads with a maximum number of mismatches of 10 % the length of the read. The sum of reads aligned within AUGUSTUS gene model predictions were used as read counts for each predicted gene. All replicates were treated separately, and read count normalization and analysis of differential expression were assessed using the edgeR package within the R statistical software suite [72] (https://cran.r-project.org/). Gene models were considered differentially expressed if they met a statistical threshold of *P* ≤ 0.01, corrected for multiple testing and a minimum change of twofold.

To test for enrichment of functional themes in lists of differentially expressed gene candidates, we utilized the annotation of *Arabidopsis* genes and their associated GO annotations. *Arabidopsis* was used instead of tomato, where there remain far fewer GO annotations than are available in *Arabidopsis*. We observed that annotations of *Hieracium* gene models to *Arabidopsis* and tomato genes generated substantial proportions of one-to-many matches. Where several distinct D18 gene models were found to match a single *Arabidopsis* gene, we multiplied the counts of the associated GO terms by the number of D18 gene models associated. We considered GO terms enriched at *P* ≤ log_10_-5. Enrichment was assessed against a background of all gene models found expressed in the ovary generated from maximum read counts across all genotypes.

### Small RNA analysis

Small RNA sequences were analysed through alignment to predicted gene models within D18 with both 5’ and 3’ boundaries extended 500 bp, allowing up to two mismatches and a microindel of 1–2 nt. Small RNA sequences were filtered to lengths of 18–25 nt, and only sequences that were observed more than five times in any one sample were retained for further analysis. Total read counts for each gene model (+/−500 bp) were normalized using the edgeR package within the R statistical software suite [72] (https://cran.r-project.org/). *MIRNA* gene prediction was completed using methods previously used in rice [73], based on detecting potential miRNA-miRNA* pairs: distinct small RNAs perfectly aligned to genomic sequences within 400 bp of each other, between which a transcribed sequence could potentially form a hairpin secondary structure.

### qRT-PCR analysis

Whole ovaries were dissected into liquid nitrogen, and RNA extracted using the RNeasy Mini Kit (Qiagen). RNA was treated with RQ1 DNase (Promega), and the reaction was cleaned up using an RNeasy Mini column (Qiagen). cDNA synthesis was carried out using 1 μg of RNA in a SuperScript III first strand synthesis reaction (Invitrogen). qRT-PCR was carried out on an RG-3000 (Corbett Research) using LightCycler 480 SYBER Green I Master Mix (Roche) with the oligonucleotides listed in Additional file 1: Table S8. The average of two biological and two technical replicates is reported using the ΔΔCt method, with *HpUBC21* as a reference gene. Genes were considered differentially expressed if they had >1.5-fold change and a paired *t*test *P* value <0.05. The validation rate of differentially expressed gene models in our transcriptome analysis using qRT-PCR was ~45 %. While low, this likely represents the difficulty in the design of allele-specific primers to D18 predicted gene models within the tetraploid plants. Oligos used for validation of the transcriptome analysis were designed from transcriptome contigs showing the best match to predicted D18 gene models. All qRT-PCR amplicons were sequenced to verify correct amplification.

### *ARGONAUTE* cloning and phylogenetic analysis

A hidden Markov model (HMM) was generated using HMMER v. 2.1 (http://hmmer.org/) from the NCBI PLN03202 plant AGO alignment. The HMM was then used to search for assembled contigs which contained AGO-related domains within *Hieracium* whole ovary transcriptomes from both the R35 and P36 genotypes. Identified AGO transcripts were amplified from cDNA libraries derived from whole ovaries at FM stage in both the R35 and P36 backgrounds. Primers used for amplification are listed in Additional file 1: Table S8. PCR products were cloned into the pCR4-TOPO vector (Invitrogen). Multiple clones were sequenced and aligned to generate a consensus sequence which was used for further phylogenetic analysis. Full-length AGO consensus protein sequences from *H. praealtum* (R35) were aligned to reference *AGO* cDNAs from *Arabidopsis* and *S. lycopersicum* using Clustal Omega [74]. An unrooted tree was constructed within MEGA6 using the maximum likelihood method based on 1000 bootstrap replicates [75]. The tree with the highest log likelihood is shown. All positions with less than 95 % site coverage were eliminated. The final dataset contained a total of 715 positions.

### In situ hybridization

Probe templates for *HpAGO1a*, *HpAGO2b* and *HpAGO5* were amplified from pCR4-TOPO cDNA clones (described above) using oligos that introduced T7 and SP6 promoters at the 5’ and the 3’ end of the amplicon, respectively (Additional file 1: Table S8). The gel purified amplicons were then used as templates for probe synthesis with the DIG RNA Labeling Kit SP6/T7 (Roche). Hybridization and visualization were performed as previously described [76].

### ARGONAUTE protein modelling

Models of the *Hieracium* HpAGO4cP36 and HpAGO4cR35 proteins were constructed by comparative (homology) modelling based on spatial restraints of the human Argonaute2 protein (accession [PDB:4OLB])[43, 77]. The HpAGO4c proteins and 4OLB protein sequences were aligned using AA-Annotator [78], followed by manual adjustments, and analysed for the dispositions of secondary structural elements [79]. The alignments were used as input parameters to build three-dimensional (3D) models within Modeller 9v8 [77]. The final 3D molecular model of both proteins was selected from 40 models that showed the lowest values of the ‘Modeller Objective Function’ and the most favourable Discrete Optimised Protein Energy (DOPE) scoring parameters [77, 80]. Stereochemical quality and overall G-factors were calculated with PROCHECK [81]. Z-score values for combined energy profiles were evaluated by Prosa2003 [82]. Structural super-positions were performed using the DeepView ‘iterative magic fit’ algorithm [83], where 760 and 751 residues (from totals of 866, 857 and 838 residues in HpAGO4cP36, HpAGO4cR35 and 4OLB, respectively) were aligned in Cα positions with root mean square deviation values of 0.68 Å and 0.72 Å for HpAGO4cP36 and HpAGO4cR35, respectively, excluding indels. Molecular graphics were generated with the PyMOL software package (http://www.pymol.org/).

## Additional files

Additional file 1: Tables S1–S8. Table S1. Summary of sequencing data across all samples. Table S2. Full list of *Arabidopsis* genes used for sexual pathway analysis. Table S3. Marker analysis in deletion mutants and polyhaploid mapping populations. Table S4. List of predicted *AGO*, *RDR* and *DCL* genes within the D18 genomic assembly. Table S5. Differentially expressed genes between MMC and FM stage ovaries in R35 and D36. Table S6. Enriched GO terms within differentially expressed genes between MMC and FM stage ovaries in R35 and D36. Table S7. List of predicted gene models with complementary small RNA and transcriptome fold changes. Table S8. List of oligonucleotides used in this study. (XLSX 142 kb)
Additional file 2: Figures S1–S6. Figure S1. Fluorescence in situ hybridization (*FISH*) of metaphase chromosomes from *m134.* Figure S2. Hierarchical clustering of sexual pathway genes in *Hieracium* ovaries. Figure S3. In situ hybridization of *HpAGO1a*, *HpAGO2b* and *HpAGO5* in *Hieracium* MMC stage ovaries. Figure S4. In situ hybridization of *HpAGO1a*, *HpAGO2b* and *HpAGO5* in *Hieracium* FM stage ovaries. Figure S5. Small RNA length distribution bar graphs. Figure S6. Coverage of small RNAs in *EXO-like* genes. (PDF 1155 kb)
Additional file 3: Supplemental data regarding transcriptome and differential expression analysis. (XLSX 4996 kb)
Additional file 4: Cloned and consensus *HpAGO* sequences in fasta format. (TXT 825 kb)
Additional file 5: Supplemental data regarding small RNA analysis. (XLSX 1496 kb)
Additional file 6: Predicted *MIRNA* genes in the D18 genome. (TXT 784 kb)
