# Supplementary material for: Generation of an integrated Hieracium genomic and transcriptomic resource enables exploration of small RNA pathways during apomixis initiation
Source: BMC Biol. 2016 Oct 6;14:86. doi: 10.1186/s12915-016-0311-0 (PMC5054587; doi:10.1186/s12915-016-0311-0)
Supplement: Additional file 2: Figures S1–S6. — Figure S1. Fluorescence in situ hybridization (FISH) of metaphase chromosomes from m134. Figure S2. Hierarchical clustering of sexual pathway genes in Hieracium ovaries. Figure S3. In situ hybridization of HpAGO1a, HpAGO2b and HpAGO5 in Hieracium MMC stage ovaries. Figure S4. In situ hybridization of HpAGO1a, HpAGO2b and HpAGO5 in Hieracium FM stage ovaries. Figure S5. Small RNA length distribution bar graphs. Figure S6. Coverage of small RNAs in EXO-like genes. (PDF 1155 kb) [file 12915_2016_311_MOESM2_ESM.pdf]

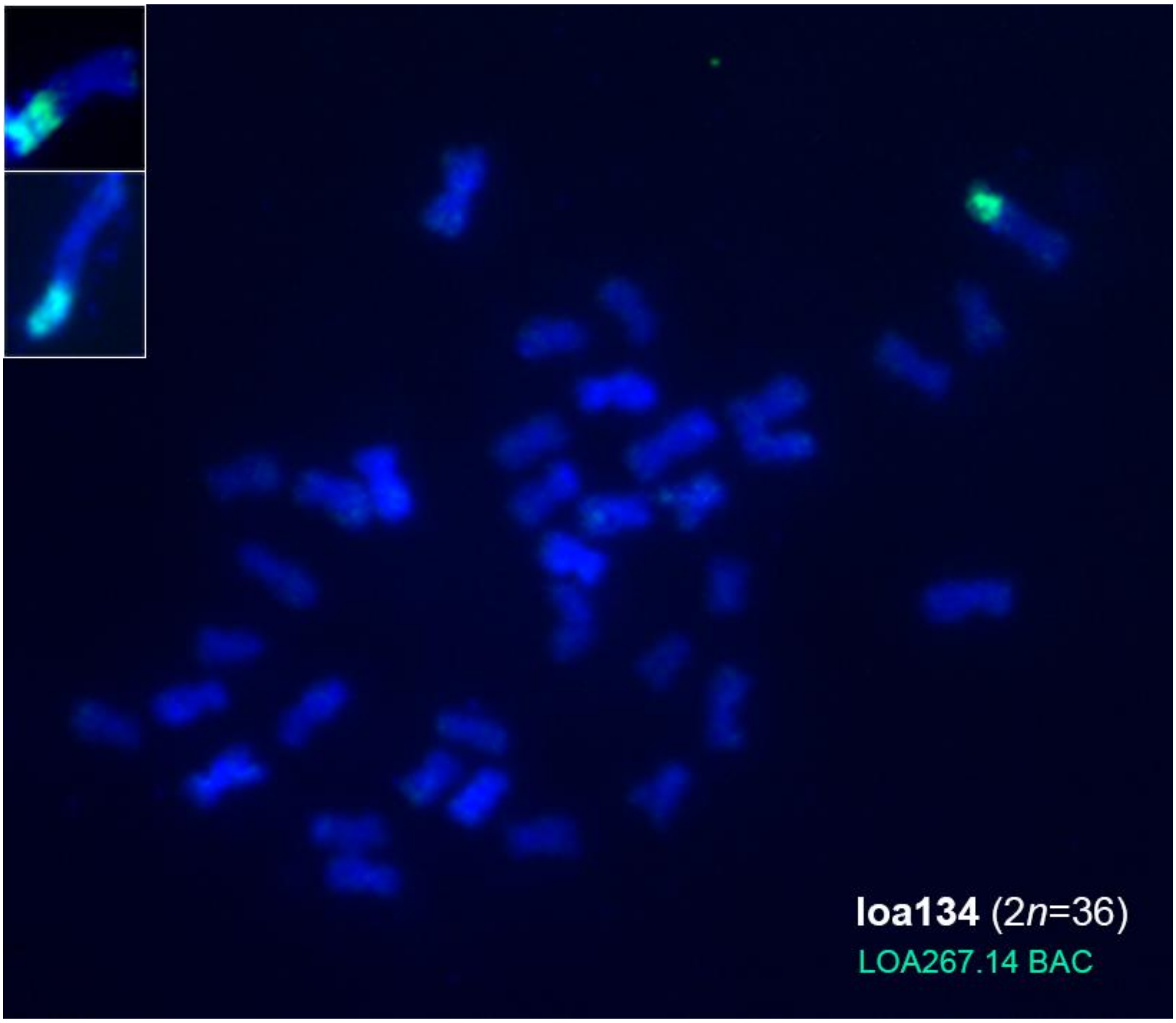

Figure S1. FISH of metaphase chromosomes from *m134*

Metaphase chromosome spreads from *m134* (loa134) plants show 36 chromosomes (2n=4x=36) as compared to its progenitor plant R35 (2n=4x-1=35) (Kotani et al., 2014). Green signal represents fluorescent in situ hybridization (FISH) using the LOA267.14 BAC probe, which is associated with the LOA locus. Insets show additional examples of LOA FISH from other spreads.

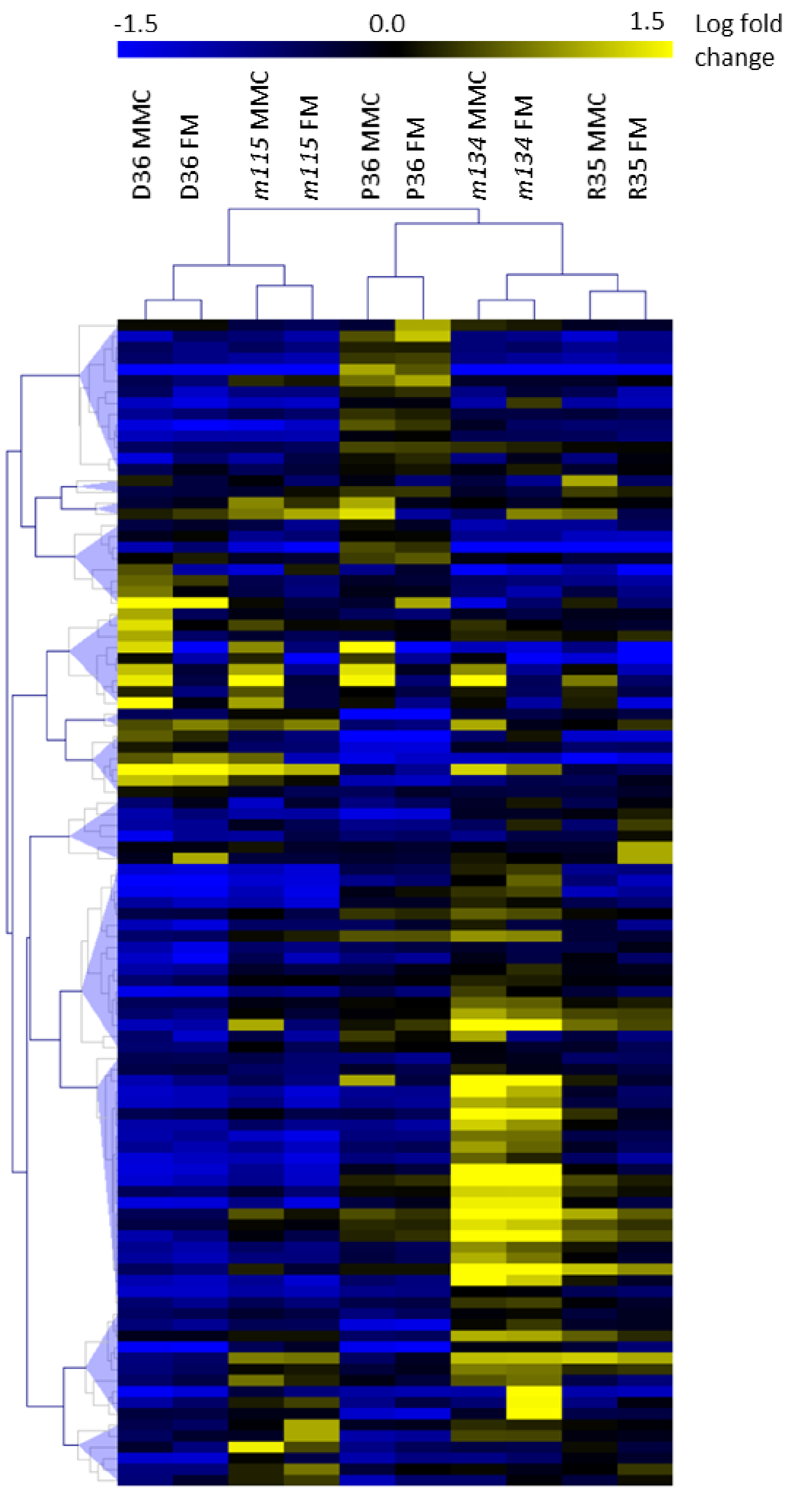

Figure S2. Hierarchal clustering of sexual pathway genes in *Hieracium* ovaries

(B) Hierarchal clustering of a subset of 105 genes with variation exceeding one standard deviation of the mean revealed that stage-specific and genotype-specific patterns could be detected.

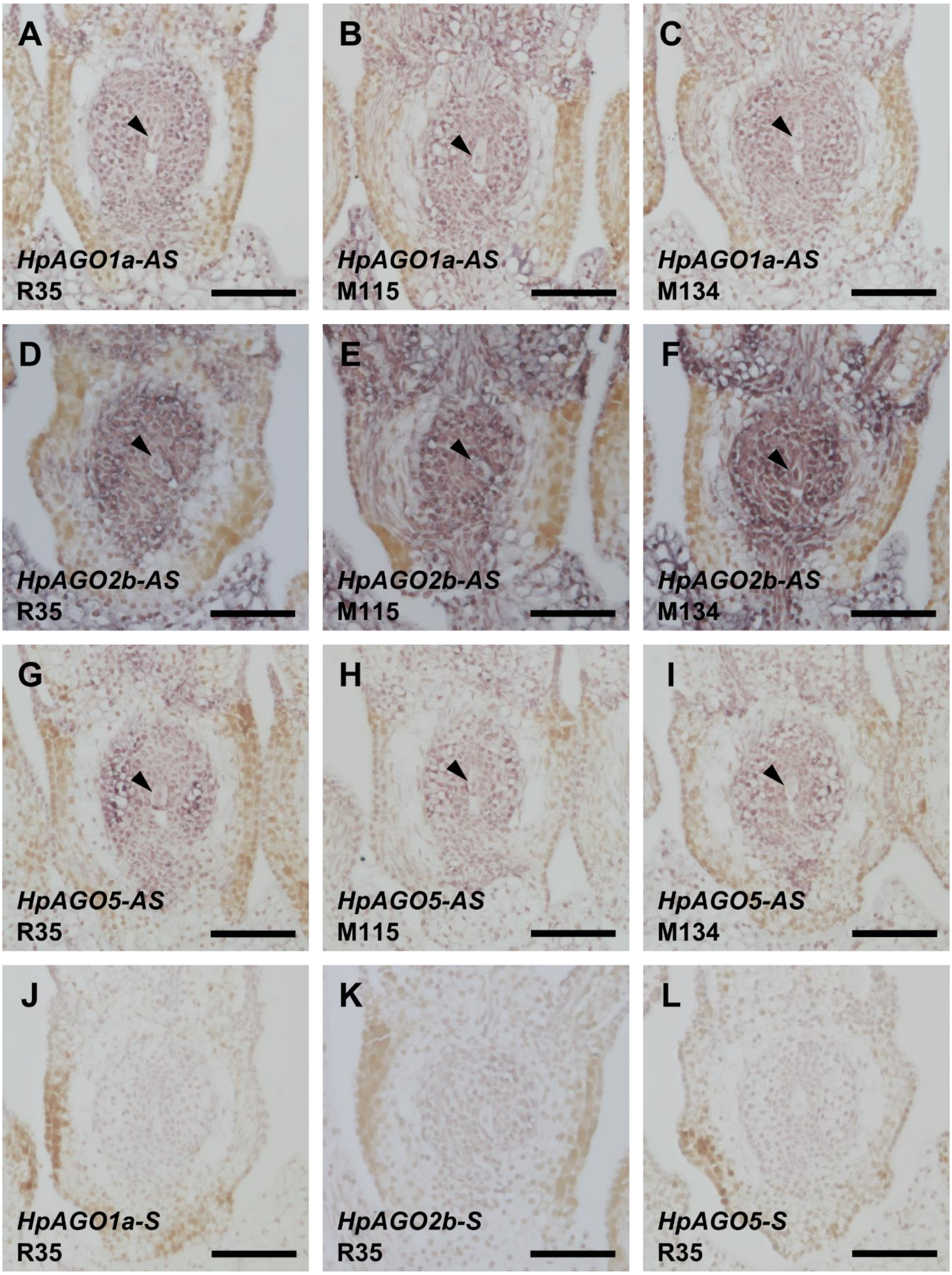

Figure S3. In situ hybridization of *HpAGO1a*, *HpAGO2b* and *HpAGO5* in *Hieracium* MMC stage ovaries

(A, B, C) Hybridization of *HpAGO1a* antisense probe to ovary sections from R35, *m115* and *m134*, respectively. (D, E, F) Hybridization of *HpAGO2b* antisense probe to ovary sections from R35, *m115* and *m134*, respectively. (G, H, I) Hybridization of

HpAGO5 antisense probe to ovary sections from R35, *m115* and *m134*, respectively. (J, K, L) Hybridization of *HpAGO1a*, *HpAGO2b* and *HpAGO5* sense probes to sections from R35 ovaries. Position of MMC is indicated by arrowheads. Scale bars = 100um.

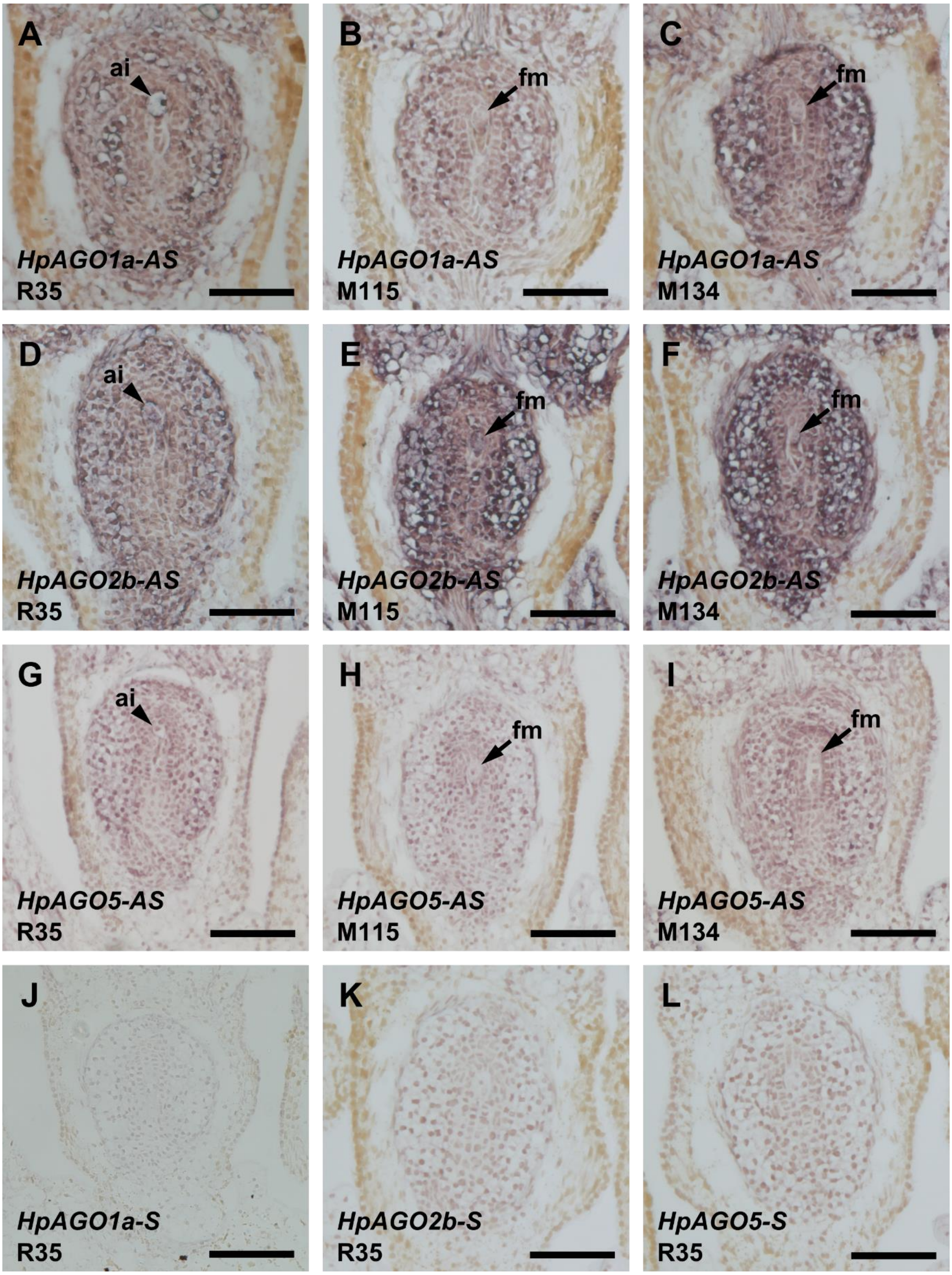

Figure S4. In situ hybridization of HpAGO1a, HpAGO2b and HpAGO5 in *Hieracium* FM stage ovaries

(A, B, C) Hybridization of *HpAGO1a* antisense probe to ovary sections from R35, *m115* and *m134*, respectively. (D, E, F) Hybridization of *HpAGO2b* antisense probe to ovary sections from R35, *m115* and *m134*, respectively. (G, H, I) Hybridization of

HpAGO5 antisense probe to ovary sections from R35, *m115* and *m134*, respectively. (J, K, L) Hybridization of *HpAGO1a*, *HpAGO2b* and *HpAGO5* sense probes to sections from R35 ovaries. The positions of AI cells within R35 sections are indicated by arrowheads. The positions of FMs in *m115* and *m134* sections are indicated by arrows. Scale bars = 100um.

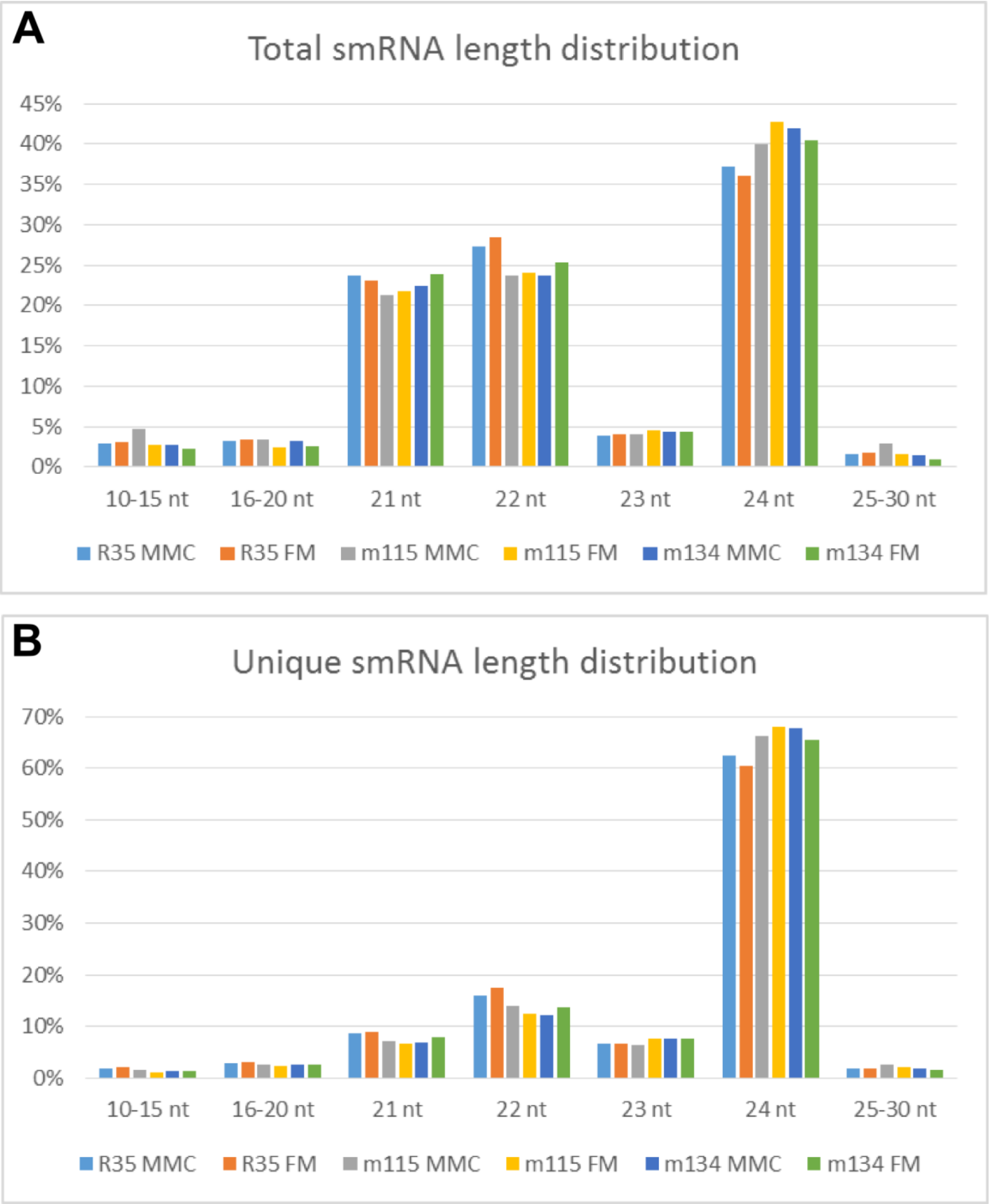

Figure S5. Small RNA length distribution bar graphs

(A) Length distribution of total sequenced small RNAs in ovaries at MMC and FM stages. (B) Length distribution of unique small RNAs in ovaries at MMC and FM stages.

A

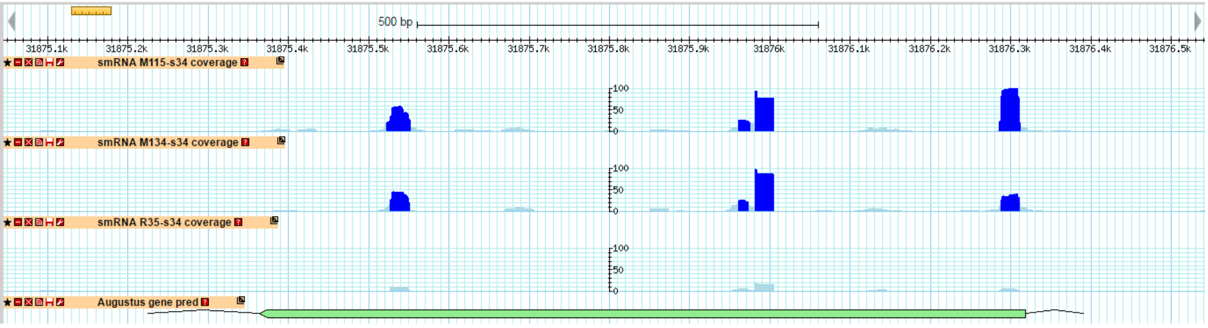

B

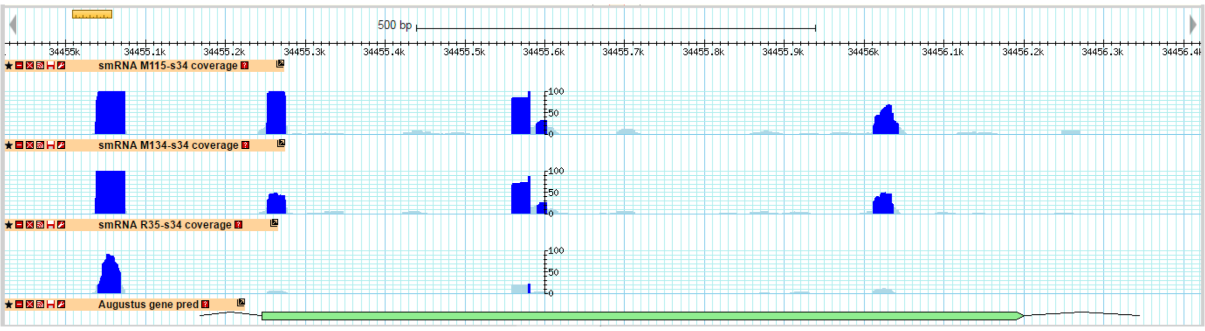

Figure S6. Coverage of small RNAs in *EXO*-like genes

A screenshot from a genome browser showing accumulation of small RNAs in the gene body of predicted *EXO*-like gene augustus-D18g-s179972.g197694 (A), and augustus-D18g-s204970.g212607 (B). Blue peaks in *m115* an *m134* coverage tracks represent increased accumulation of 24nt small RNAs in these backgrounds. Gene prediction exons are indicated in green.
